# Human Trace Elements, Gut Microbiota, and Alzheimer's Disease: Insights From Multistage Mendelian Randomization Analysis

**DOI:** 10.1002/fsn3.70706

**Published:** 2025-08-12

**Authors:** Yujian Li, Hao Lin, Kexin Liu, Xuan Kan

**Affiliations:** ^1^ School of Public Health Tianjin Medical University Tianjin China; ^2^ General Hospital of Tianjin Medical University Tianjin China; ^3^ Medical School of Tianjin University Tianjin China

**Keywords:** Alzheimer's disease, gut microbiota, mediation effect, Mendelian randomization, trace elements

## Abstract

Alzheimer's disease (AD) is a prevalent neurodegenerative disorder characterized by progressive cognitive decline that ultimately impairs independent living. Previous studies have identified associations between certain trace elements or gut microbiota and AD, but few have explored the interplay among all three. This study aimed to elucidate the relationship between 17 human trace elements and AD using Mendelian randomization (MR) and multivariate MR (MVMR) analysis. In addition, the potential mediating role of 412 gut microbiota in the relationship between trace elements and AD was investigated using mediation MR analysis. MR analysis revealed the genetic causal associations of copper (Odds Ratio [OR] = 1.291, 95% Confidence Interval [CI]: 1.075–1.551) and carotene (OR = 2.805, 95% CI: 1.054–7.466) with AD. MVMR analysis further supported that copper (OR = 1.262, 95% CI: 1.084–1.468) and carotene (OR = 4.550, 95% CI: 1.245–16.631) were independent risk factors for AD. Mediation MR analysis indicated that increased copper levels enhance lipid biosynthesis pathways (OR = 1.125, 95% CI: 1.046–1.210), contributing to a higher AD risk (OR = 1.822, 95% CI: 1.036–3.206), with a mediation effect of 27.636%. Elevated carotene levels were associated with reduced pentose phosphate pathway activity and decreased abundance of f_Prevotellaceae.g_Paraprevotella, both of which were linked to increased AD risk, with mediation effects of 25.527% and 32.140%, respectively. This study highlights copper and carotene as potential targets for novel AD interventions and underscores the complex interplay between trace elements, gut microbiota, and neurodegenerative diseases.

## Graphical abstract

This study offers a comprehensive investigation into the intricate relationships between human trace elements, gut microbiota, and Alzheimer's disease (AD) using advanced Mendelian randomization (MR) methodologies, addressing critical gaps in the current understanding of AD pathogenesis.

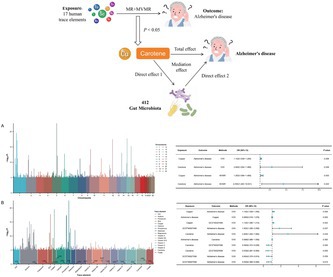

**Abbreviations**
AD: Alzheimer's disease
CI: confidence interval
GCST90027490: NAGLIPASYN.PWY..lipid.IVA.biosynthesis
GCST90027506: PENTOSE.P.PWY..pentose.phosphate.pathway
GCST90027699: k_Bacteria.p_Bacteroidetes.c_Bacteroidia.o_Bacteroidales.f_Prevotellaceae.g_Paraprevotella
GWAS: Genome‐wide association study
IVs: instrumental variables
IVW: inverse variance weighted
LD: linkage disequilibrium
MR: Mendelian randomization
MVMR: multivariate Mendelian randomization
OR: odds ratio

## 1 Introduction

Alzheimer's disease (AD) is a prevalent neurodegenerative disorder that primarily affects the elderly population (Scheltens et al. 2021). Characterized by a progressive decline in cognitive function, AD ultimately leads to an inability to perform daily activities independently (Ferrari and Sorbi 2021). The disease imposes a significant burden on both patients and society, manifesting in substantial healthcare costs and emotional distress for families (*Alzheimer's and Dementia: The Journal of the Alzheimer's Association* 2024). With the global population aging, the prevalence of AD is projected to triple by 2050 (Mead and Fox 2023). Current therapeutic strategies are predominantly focused on symptom management, with limited options available for effective prevention or cure, highlighting the urgent need for research aimed at identifying novel risk factors and therapeutic targets for Alzheimer's disease (Ossenkoppele et al. 2022; Khan et al. 2020).

Recent studies have suggested that certain trace elements in the human body might be associated with the development and progression of AD (Yan et al. 2021; Li, Li, et al. 2023). However, the specific mechanisms underlying these associations remain largely unclear (Huang 2023; Doroszkiewicz et al. 2023). Although previous research has indicated potential links between various trace elements and AD, findings have often been inconsistent and inconclusive (Kawahara et al. 2023; Botchway et al. 2023; Drew 2017). In addition, previous studies have focused more on commonly known elements such as copper, zinc, and iron, while research on other trace elements is limited (Nikseresht et al. 2019; Posadas et al. 2022). This study aims to address these gaps by employing a comprehensive approach that includes Mendelian randomization (MR) analysis, multivariate MR (MVMR) analysis, and mediation MR analysis to explore the potential relationships between 17 trace elements and AD. By doing so, this research seeks to provide a more robust understanding of the role that trace elements may play in AD pathogenesis.

The methodological framework of this study is designed to leverage the strengths of multiple analytical techniques. Mendelian randomization utilizes genetic variants as instrumental variables to infer causal relationships, thereby mitigating confounding factors common in observational studies (Larsson et al. 2023). The gut microbiota interacts with target organs such as the kidneys and liver through metabolic and immune pathways, thereby playing a crucial role in the onset and progression of various diseases (Fan et al. 2024; Huang, Li, et al. 2024; Li et al. 2025). Additionally, growing evidence from studies on the gut–brain axis has found that the role of gut microbiota in the pathogenesis of metabolic disorders and AD (Wang et al. 2019; Zhu et al. 2022). Based on this, mediation MR analysis is incorporated to examine whether gut microbiota mediate the relationship between trace elements and AD (Carter et al. 2021). This multi‐faceted approach enhances the reliability and validity of the findings, offering a comprehensive perspective on the subject.

Despite previous efforts, few studies have systematically explored the potential causal links between a broad range of trace elements and Alzheimer's disease using genetically informed methodologies. The primary objective of this study is to elucidate the relationships between 17 specific trace elements and AD. By employing traditional MR, MVMR, and mediation MR, this research aims to identify which trace elements are significantly associated with AD and to explore the potential mechanisms underlying these associations. Besides, the study also seeks to determine whether gut microbiota serve as mediators in the relationship between trace elements and AD. This multi‐layered approach represents a novel intersection of nutritional epidemiology, microbial science, and neurogenetics. This investigation is expected to yield insights that could inform the development of novel preventive and therapeutic strategies for AD.

In summary, this study addresses a critical gap in the current understanding of AD by focusing on the roles of trace elements and their potential interactions with gut microbiota. The use of various forms of MR provides a robust framework for exploring these complex relationships. The findings from this research have the potential to contribute significantly to the field of AD, paving the way for future investigations and clinical applications that may ultimately alleviate the burden of this devastating disease.

## 2 Methods

**FIGURE 1:**
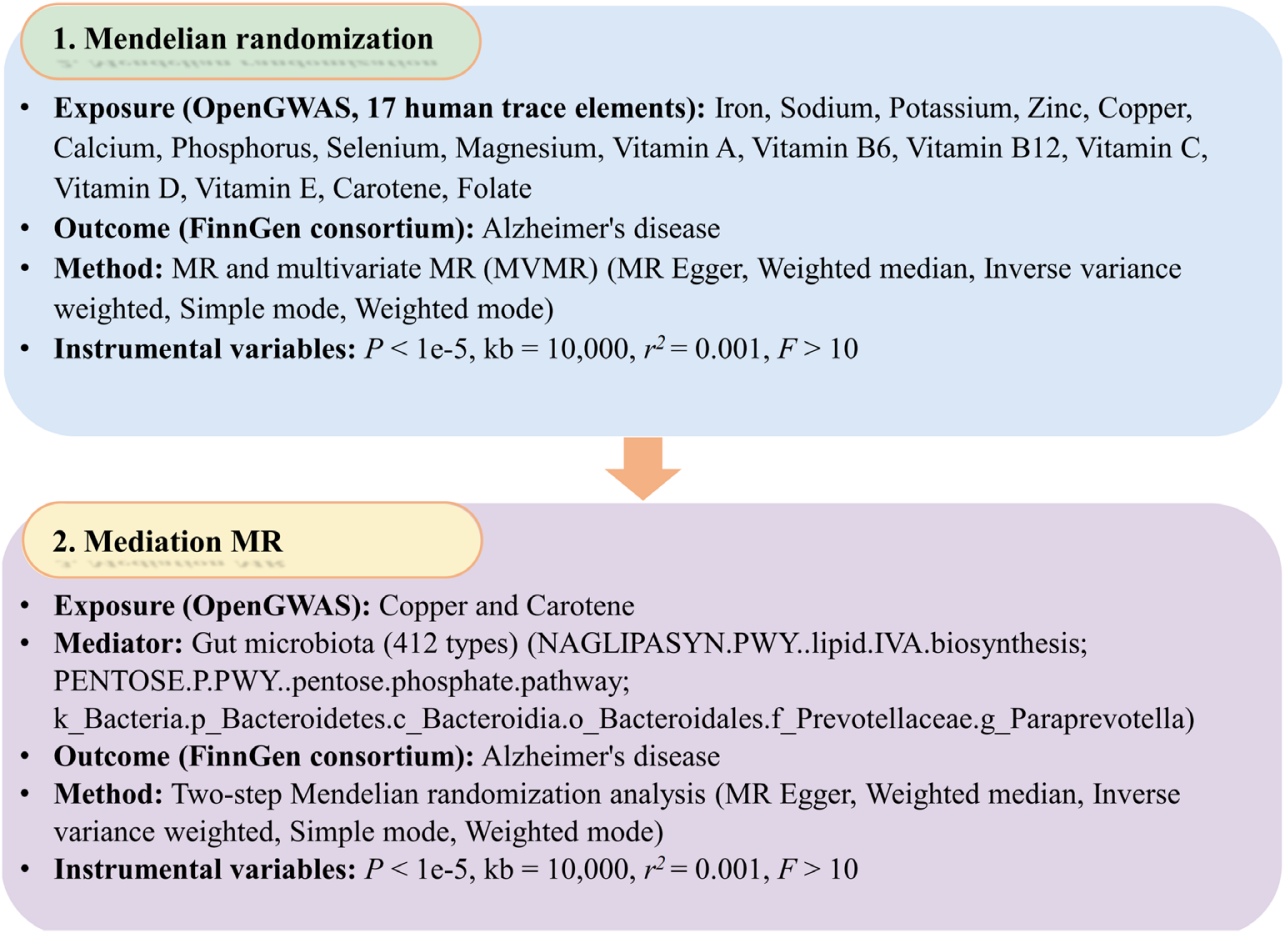
The flow chart. The study was divided into two phases. The first phase explored the genetic causality between the 17 human trace elements and AD with the help of MR analysis and MVMR analysis. The second phase explored the role of gut microbiota and its related pathways in the influence of trace elements on AD with the help of mediation MR analysis. AD, Alzheimer's disease; MR, Mendelian randomization; MVMR, Multivariate Mendelian randomization.

Figure 1 presents the overall research design and analytical workflow. All data analyses were performed using R software (4.2.3), and the detailed materials and methods section are outlined below.

### 2.1 MR Analysis and MVMR Analysis

Considering that MR analysis can better reduce the influence of confounding factors on causal association studies (Yang et al. 2022), we used MR to analyze the causal relationships between exposure (human trace elements) and outcome (AD) in order to obtain more accurate results. The primary analytical method of MR used was the inverse variance weighted (IVW), supplemented by four additional methods (MR Egger, weighted median, simple mode, and weighted mode) to enhance result robustness (Li, Wang, et al. 2023). MVMR analysis was subsequently conducted to further clarify the independent causal relationship between the positive findings of trace elements and AD (Sanderson 2021). Genetic causality between exposure and outcome was considered to exist when *p* < 0.05 for the findings.

Genome‐wide association study (GWAS) data for the 17 human trace elements (as exposures) were obtained from the OpenGWAS database (https://gwas.mrcieu.ac.uk/), whereas GWAS data for AD (as outcome) were obtained from the FinnGen consortium (https://storage.googleapis.com/finngen‐public‐data‐r10/summary_stats/finngen_R10_AD_U_EXMORE.gz). Instrumental variables (IVs) between human trace elements and AD were selected based on the following criteria: (1) IVs and exposure were correlated (*p* < 1e‐5); (2) linkage disequilibrium (LD) was excluded (*r* ^2^ > 0.001, kb = 10,000); (3) IVs had sufficient strength of association with exposure (*F* > 10); and (4) confounding factors of IVs were eliminated with the help of the PubMed database (https://pubmed.ncbi.nlm.nih.gov/) and OpenGWAS database (Wang et al. 2022).

### 2.2 Mediation MR Analysis

To further investigate how human trace elements affect the onset and progression of AD, we conducted the two‐step mediation MR analysis to assess whether gut microbiota mediate these effects. The exposure for the mediation MR analysis was the human trace element that was the positive result of the MR analysis described above, and the outcome was AD. The gut microbiota data, used as a mediator, were derived from a previous study (https://dutchmicrobiomeproject.molgeniscloud.org/) and contained a total of 412 gut microbiota and associated pathways (Lopera‐Maya et al. 2022). IVs were screened for *p* < 1e‐5, kb = 10,000, *r* ^2^ > 0.001, *F* > 10, and confounders were excluded.

### 2.3 Sensitivity Analysis

To enhance the credibility of the MR results, we conducted several sensitivity analyses, including heterogeneity test, horizontal multiple validity test, and leave‐one‐out sensitivity analysis (Huang et al. 2023). For the positive results, the heterogeneity test among the IVs was first used. When *p* > 0.05 indicated no significant heterogeneity among the IVs, the results of the MR analysis were presented using a fixed‐effects model, and vice versa using a random‐effects model. Secondly, leave‐one‐out sensitivity analysis was used to test the effect of individual IVs on the overall results of the MR analysis. Finally, since the MR Egger method takes into account the intercept term when performing the regression analysis, the horizontal multiple validity test will compare the MR Egger method with the IVW method to verify whether there is horizontal multiple validity between IVs. When *p* > 0.05 was interpreted as no horizontal multiple validity between IVs, the IVW method was used as the result of the MR analysis, and on the contrary, the MR Egger method was used as the result of the MR analysis (Chen et al. 2022).

## 3 Results

### 3.1 MR Analysis

**FIGURE 2:**
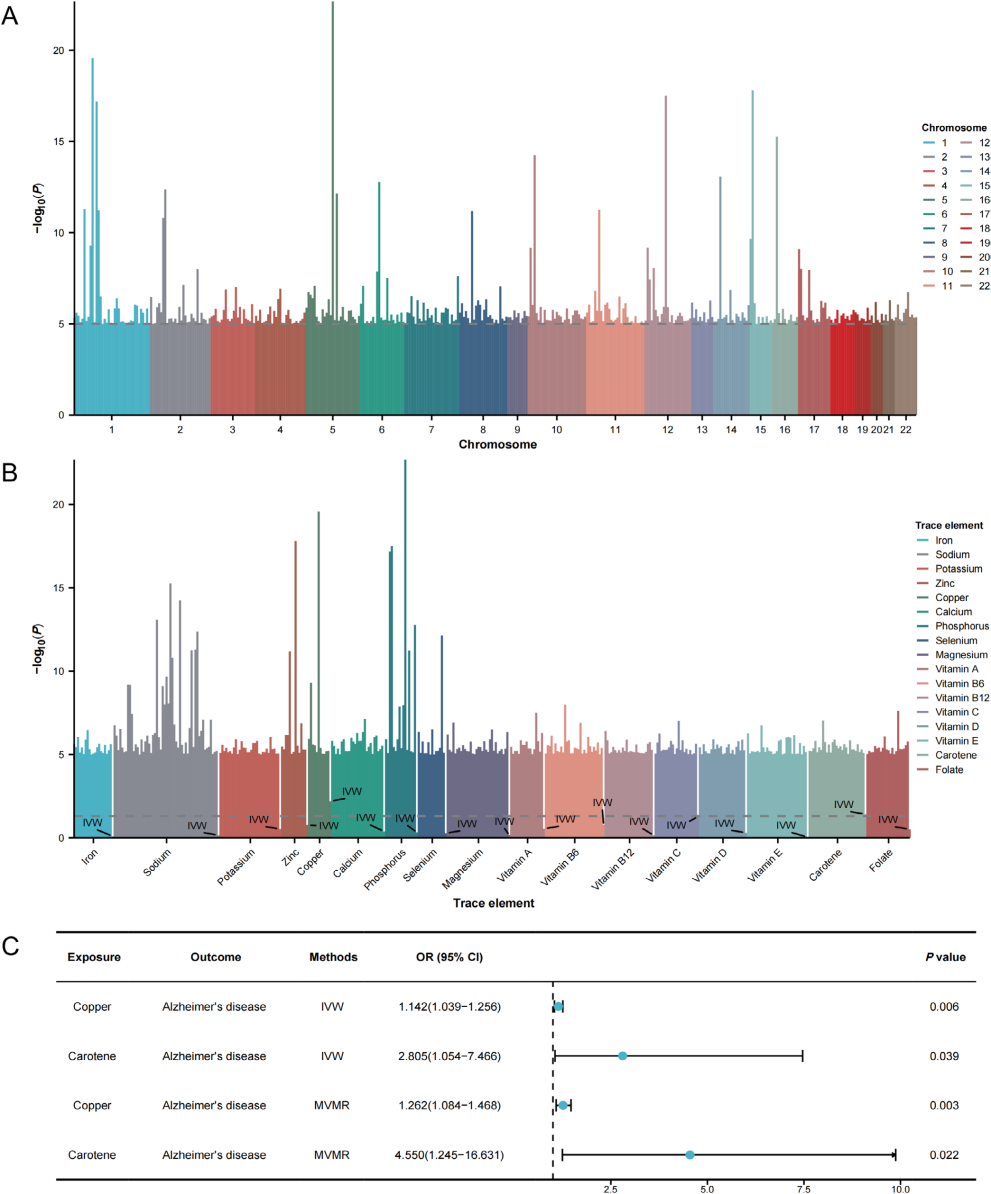
MR analysis and MVMR analysis. (A) Manhattan plot of SNPs included in MR analysis. (B) Manhattan plot of MR analysis results. (C) Forest plot of MR analysis and MVMR analysis. MR, Mendelian randomization; MVMR, Multivariate Mendelian randomization; SNPs, Single nucleotide polymorphisms.

The exposure data for the 17 human trace elements encompassed participants ranging from thousands to hundreds of thousands, while the AD outcome dataset included 184,190 participants. MR analysis identified genetic causal associations between two human trace elements and AD: copper (Odds Ratio [OR] = 1.291, 95% Confidence Interval [CI]: 1.075–1.551) and carotene (OR = 2.805, 95% CI: 1.054–7.466). Copper's GWAS data included 2603 participants, while carotene's data included 64,979 participants. After rigorous screening of the IVs, 11 single nucleotide polymorphisms (SNPs) were finally retained for copper and 29 SNPs for carotene in the MR analysis (Figure 2, Tables S1–S3). To further examine whether copper and carotene independently contribute to AD, we performed MVMR analysis. The results of the analysis similarly supported that copper (OR = 1.262, 95% CI: 1.084–1.468) and carotene (OR = 4.550, 95% CI: 1.245–16.631) were independent risk factors for AD (Figure 2C).

### 3.2 Mediation MR Analysis

**FIGURE 3:**
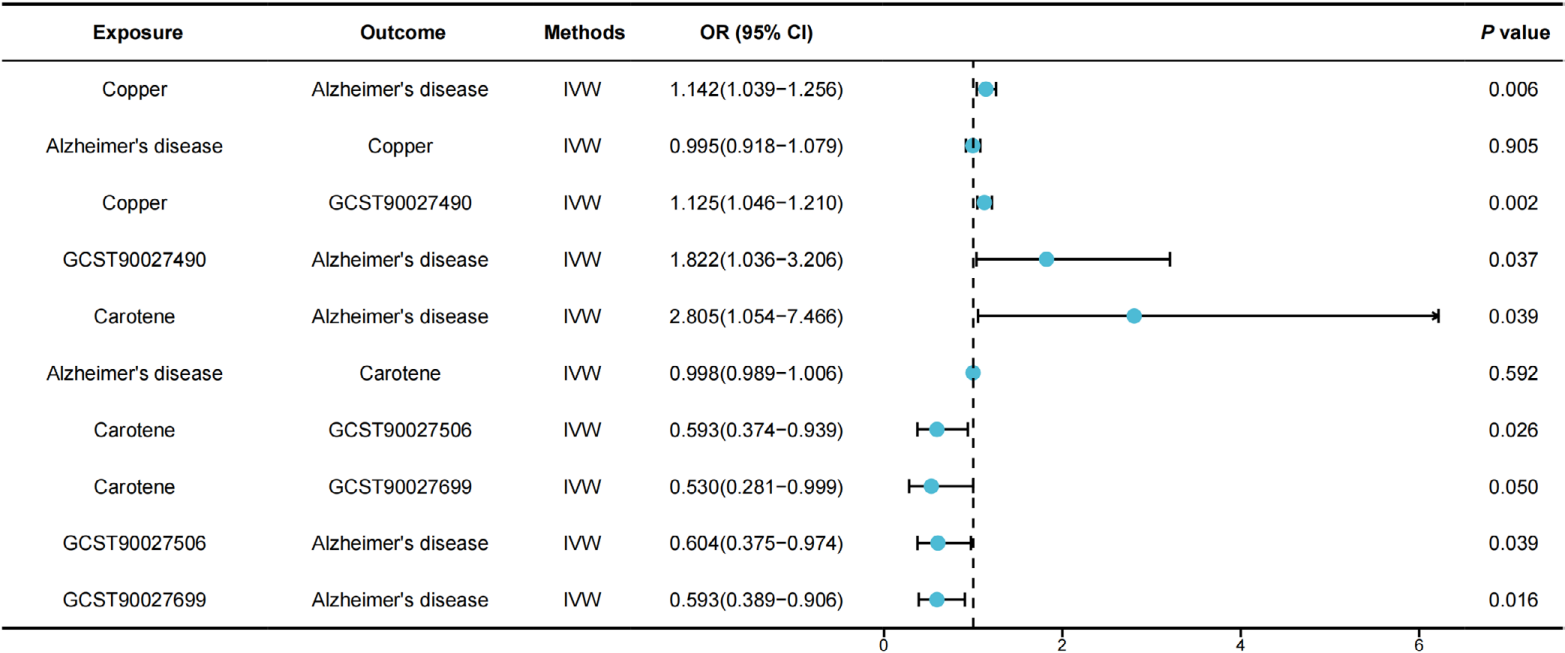
Forest plot of mediation MR analysis (trace elements, gut microbiota, and AD). AD, Alzheimer's disease; MR, Mendelian randomization.

Based on the positive findings from the MR analysis, copper and carotene were selected as exposures for the mediation MR analysis. Data on the 412 gut microbiota and associated pathways used as mediators were obtained from 7738 participants (Table S1). Reverse MR analysis showed no significant association between copper and carotene with AD were negative. Mediation MR analysis identified 3 out of 412 gut microbiota and related pathways as mediating regulatory roles in copper and carotene affecting the development of AD. A total of 9 SNPs satisfying the screening criteria were matched between copper and GCST90027490, while 10 SNPs were matched between GCST90027490 and AD. Increased levels of copper in the organism were associated with increased expression of GCST90027490 (OR = 1.125, 95% CI: 1.046–1.210), which in turn increased the risk of AD (OR = 1.822, 95% CI: 1.036–3.206), of which the mediating role of GCST90027490 accounted for 27.636%. Negative genetic causality was observed in carotene with GCST90027506 (OR = 0.593, 95% CI: 0.374–0.939) and GCST90027699 (OR = 0.530, 95% CI: 0.281–0.999), whereas the negative genetic causality was found between GCST90027506 (OR = 0.604, 95% CI: 0.375–0.974), GCST90027699 (OR = 0.593, 95% CI: 0.389–0.906) and AD. Increased levels of carotene in the body led to decreased expression of GCST90027506 and GCST90027699, which increased the risk of AD, with mediating effects accounting for 25.527% and 32.140%, respectively (Table 1, Figure 3, Tables S2 and S3).

**TABLE 1:** The mediation effect of trace element on Alzheimer's disease via gut microbiota.

Trace element—Gut microbiota—Alzheimer's disease	Total effect	Direct effect 1	Direct effect 2	Direct effect	Mediation effect	Mediated proportion (%) (95% CI)
	*β*	*β*	*β*	*β*	*β* (95% CI)	
Copper—GCST90027490—AD	0.255	0.118	0.600	0.185	0.071 (−0.102, 0.244)	27.636 (−40.095, 95.366)
Carotene—GCST90027506—AD	1.031	−0.523	−0.503	0.768	0.263 (0.090, 0.437)	25.527 (8.717, 42.338)
Carotene—GCST90027699—AD	1.031	−0.635	−0.522	0.700	0.331 (0.097, 0.566)	32.140 (9.415, 54.865)

Abbreviations: AD, Alzheimer's disease; GCST90027490, NAGLIPASYN.PWY..lipid.IVA.biosynthesis; GCST90027506, PENTOSE.P.PWY..pentose.phosphate.pathway; GCST90027699, k_Bacteria.p_Bacteroidetes.c_Bacteroidia.o_Bacteroidales.f_Prevotellaceae.g_Paraprevotella.

### 3.3 Sensitivity Analysis

The test for horizontal pleiotropy showed *p* > 0.05, indicating that no evidence of horizontal pleiotropy was observed among the IVs in this study. Since the heterogeneity test for a few outcomes showed the presence of heterogeneity, we corrected for these outcomes using the random effects model, and the IVW results still held (Figures S1 and S2).

## 4 Discussion

AD poses economic, emotional, and social burdens, and the lack of effective treatment highlights the necessity for research aimed at uncovering novel risk factors and potential therapeutic targets (Ding et al. 2022). Our study leverages a comprehensive approach, utilizing MR analysis, MVMR analysis, and mediation MR analysis to investigate the association between 17 human trace elements, gut microbiota, and AD. This multifaceted methodology enhances the reliability and robustness of our findings. Our key results indicate that copper and carotene are independent risk factors for AD, and that gut microbiota plays a mediating role in this association. These findings not only highlight the potential of copper and carotene as biomarkers for AD risk, but also suggest new avenues for therapeutic intervention targeting the gut‐brain axis.

MR analysis provided robust evidence supporting the genetic causal relationships between copper (OR = 1.291, 95% CI: 1.075–1.551) and carotene (OR = 2.805, 95% CI: 1.054–7.466) with AD. MVMR analysis further corroborated these findings, establishing copper (OR = 1.262, 95% CI: 1.084–1.468) and carotene (OR = 4.550, 95% CI: 1.245–16.631) as independent genetic risk factors for AD. These results reinforce the significance of copper and carotene in AD pathogenesis. The genetic causal relationships identified in this study highlight the importance of understanding the underlying mechanisms through which copper and carotene influence AD. A study by Huang D et al. proposed that copper accumulation in mitochondria increases the risk of developing AD, which is consistent with the results of the present study (Huang, Chen, et al. 2024). While other studies have found copper to be associated with Aβ plaque formation and oxidative stress, which may promote neurodegeneration (Ho et al. 2022; Sun et al. 2022). In addition, Patel and Aschner (2021) found that neurotoxins of metallic elements such as copper may also have a correlation with AD. On the contrary, previous studies have proposed a possible protective effect against AD due to carotene being associated with antioxidants, contradicting our results (Yi et al. 2023; Power et al. 2022). This discrepancy may stem from differences in carotene levels, isomeric forms, or interactions with other metabolic factors. Future research should delve into the mechanistic pathways by which copper and carotene contribute to AD. Additionally, extending MR analysis to other nutrients and diseases could provide valuable insights into their genetic causal relationships. The interplay between copper, carotene, and genetic factors in other health conditions should also be explored to understand their broader implications on health.

Mediation MR analysis revealed significant indirect effects through the gut microbiota. Elevated copper levels lead to increased lipid biosynthesis pathways (OR = 1.125, 95% CI: 1.046–1.210), which in turn increase AD risk (OR = 1.822, 95% CI: 1.036–3.206), with a mediation effect of 27.636%. In contrast, carotene exhibited negative genetic causal relationships with the pentose phosphate pathway (OR = 0.593, 95% CI: 0.374–0.939) and f_Prevotellaceae.g_Paraprevotella (OR = 0.530, 95% CI: 0.281–0.999), both of which were negatively associated with AD risk (pentose phosphate pathway: OR = 0.604, 95% CI: 0.375–0.974; f_Prevotellaceae.g_Paraprevotella: OR = 0.593, 95% CI: 0.389–0.906). Increased carotene levels led to reduced pentose phosphate pathway activity and decreased f_Prevotellaceae.g_Paraprevotella abundance, thereby elevating AD risk, with mediation effects of 25.527% and 32.140%, respectively. These findings underscore the critical role of gut microbiota in mediating the effects of copper and carotene on AD. Tcw et al. (Tcw et al. 2022) showed that stabilization of apolipoprotein E ε4 is important for AD, and therefore the risk of AD is likely to increase when abnormal lipid biosynthesis is enhanced. A previous systematic review showed that Prevotellaceae flora abundance was lower in Parkinson's patients than in normal control populations (Heravi et al. 2023), suggesting that reduced Prevotellaceae flora abundance may increase the risk of developing neurodegenerative diseases. Future research should focus on elucidating the specific relationships among lipid biosynthesis pathways, the pentose phosphate pathway, f_Prevotellaceae.g_Paraprevotella, and AD. Additionally, Prevotellaceae has also been found to be associated with sepsis and oral cancer (Zhang et al. 2019; Luo et al. 2023). Furthermore, metabolomic studies have highlighted significant metabolic dysregulation during AD progression, including inflammation and impaired energy metabolism (Li et al. 2022; Luo et al. 2019). These observations collectively support the central role of the gut–brain axis in the pathophysiology of AD and point to potential multi‐target therapeutic strategies that address both neurodegeneration and metabolic dysfunction (Zhang et al. 2024).

Sensitivity analysis indicated no evidence of horizontal pleiotropy (*p* > 0.05), enhancing the credibility of our findings. The absence of horizontal pleiotropy suggests that the IVs used in this study are valid and not influenced by confounding factors. The robustness of our results was further supported by the consistency of the IVW results, even after adjusting for heterogeneity using random‐effects models. In future studies, the selection criteria for IVs and their impact on the stability of MR analysis results should be carefully considered. The importance of different models (fixed‐effects vs. random‐effects) should also be emphasized when interpreting sensitivity analysis results. Sensitivity analysis should be applied in other epidemiological studies to ensure the robustness and validity of their findings.

This study has several limitations that should be acknowledged. Firstly, the absence of wet lab experiments limits the ability to validate the findings through direct biological assays. Secondly, although the sample size was generally large, it may still be insufficient to fully capture the spectrum of variability in the population, potentially affecting the generalizability of the results. Additionally, batch effects between different datasets could introduce biases that are not entirely mitigated by the statistical methods employed. These limitations suggest that further studies, including larger cohorts and experimental validations, are necessary to confirm and extend our findings.

## 5 Conclusions

In conclusion, this study employs a comprehensive approach combining MR, MVMR, and mediation MR to elucidate the roles of copper and carotene as independent risk factors for AD. Our findings suggest that these elements might influence disease progression through interactions with the gut microbiota, specifically lipid biosynthesis pathways and the pentose phosphate pathway. These insights provide a theoretical basis for novel intervention strategies targeting nutritional and microbial factors in AD. Future research should focus on experimental validation and exploring the complex interplay between nutrients, gut microbiota, and neurodegenerative diseases.

## Author Contributions

**Yujian Li:** conceptualization (equal), data curation (equal), methodology (equal), writing – original draft (equal). **Hao Lin:** conceptualization (equal), data curation (equal), methodology (equal), writing – original draft (equal). **Kexin Liu:** conceptualization (equal), data curation (equal), funding acquisition (equal), writing – review and editing (equal). **Xuan Kan:** writing – review and editing (equal).

## Conflicts of Interest

The authors declare no conflicts of interest.

## Supporting information


**Figure S1:** Funnel plot, Scatterplot and Leave‐one‐out test of MR analysis and mediation MR analysis (copper, gut microbiota, and Alzheimer's disease).

**Figure S2:** Funnel plot, Scatterplot and Leave‐one‐out test of MR analysis and mediation MR analysis (carotene, gut microbiota, and Alzheimer's disease).

**Table S1:** Baseline information table for exposure, mediator and outcome.
**Table S2:** Screening results of single nucleotide polymorphism.
**Table S3:** Mendelian randomization analysis and sensitivity analysis of genetic causality between trace element, gut microbiota and Alzheimer's disease.

## Data Availability

The datasets analyzed in this study are from (1) OpenGWAS database (https://gwas.mrcieu.ac.uk/) (accession nos. ukb‐b‐20447, bbj‐a‐43, ukb‐b‐17881, ieu‐a‐1079, ieu‐a‐1073, ukb‐b‐8951, bbj‐a‐45, ieu‐a‐1077, ukb‐b‐7372, ukb‐b‐9596, ukb‐b‐7864, ukb‐b‐19524, ukb‐b‐19390, ukb‐b‐18593, ukb‐b‐6888, ukb‐b‐16202, ukb‐b‐11349); (2) FinnGen consortium (https://storage.googleapis.com/finngen‐public‐data‐r10/summary_stats/finngen_R10_AD_U_EXMORE.gz) (accession nos. finngen_R10_AD_U_EXMORE); and (3) PubMed (https://dutchmicrobiomeproject.molgeniscloud.org/) (accession nos. PMID: 35115690).
