# Supplementary figures and images for: Human Trace Elements, Gut Microbiota, and Alzheimer's Disease: Insights From Multistage Mendelian Randomization Analysis
Source: Food Sci Nutr. 2025 Aug 12;13(8):e70706. doi: 10.1002/fsn3.70706 (PMC12340980; doi:10.1002/fsn3.70706)

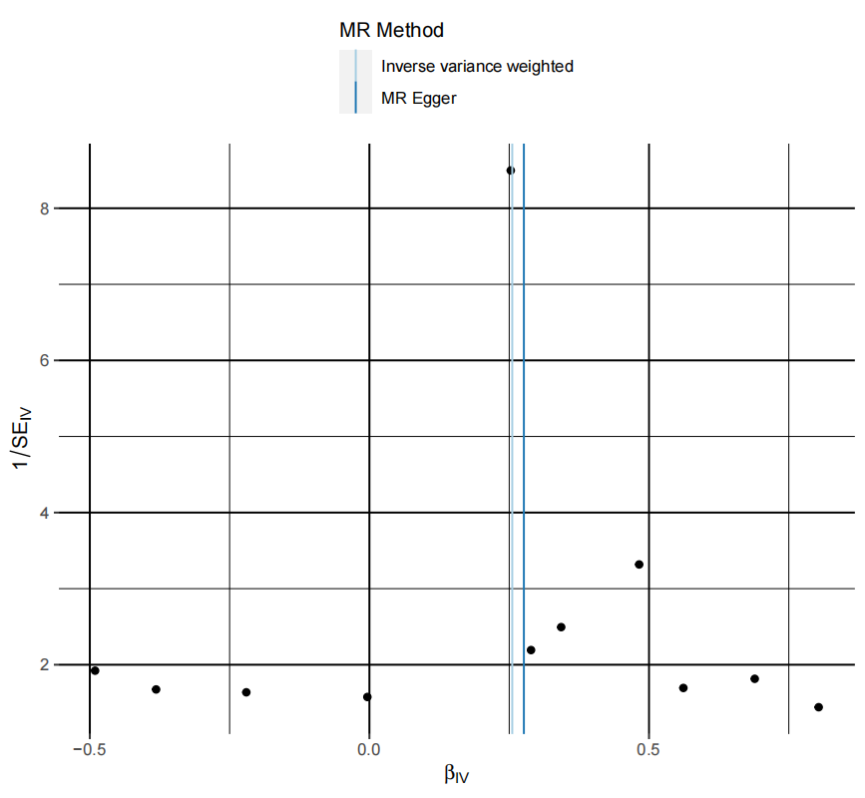


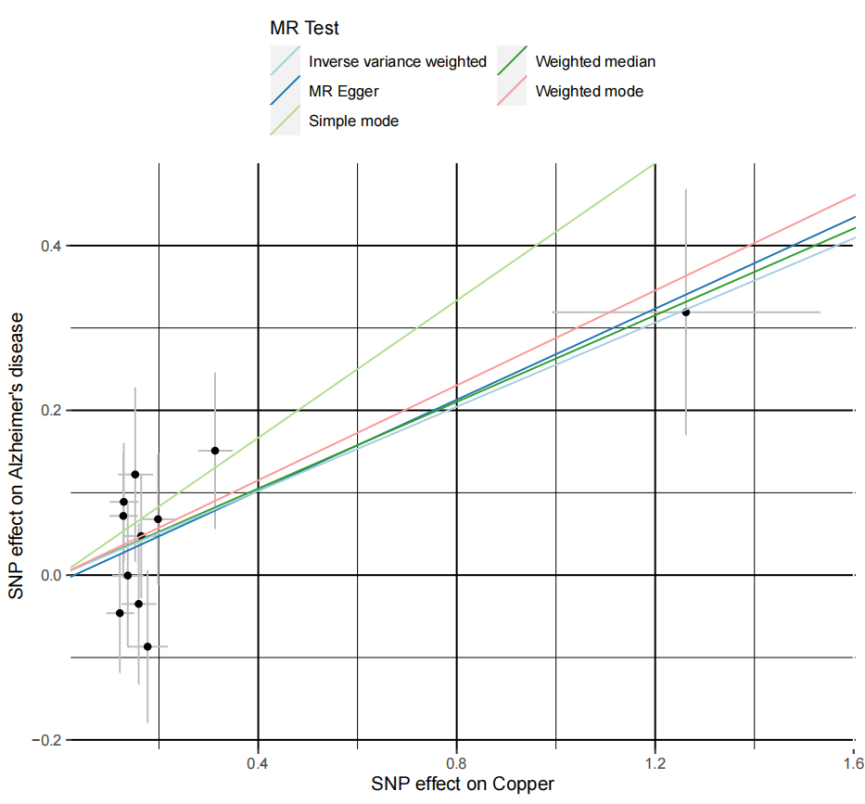


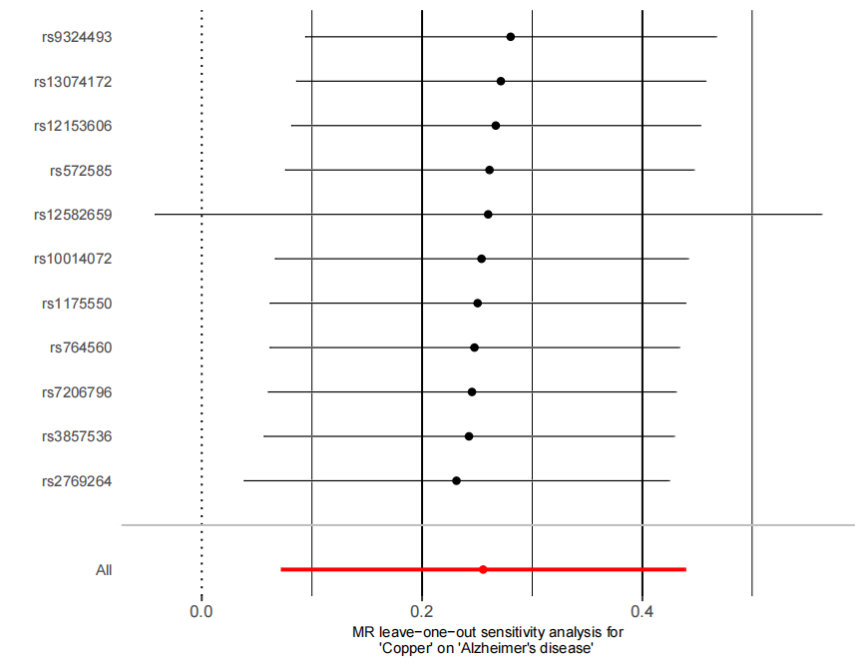


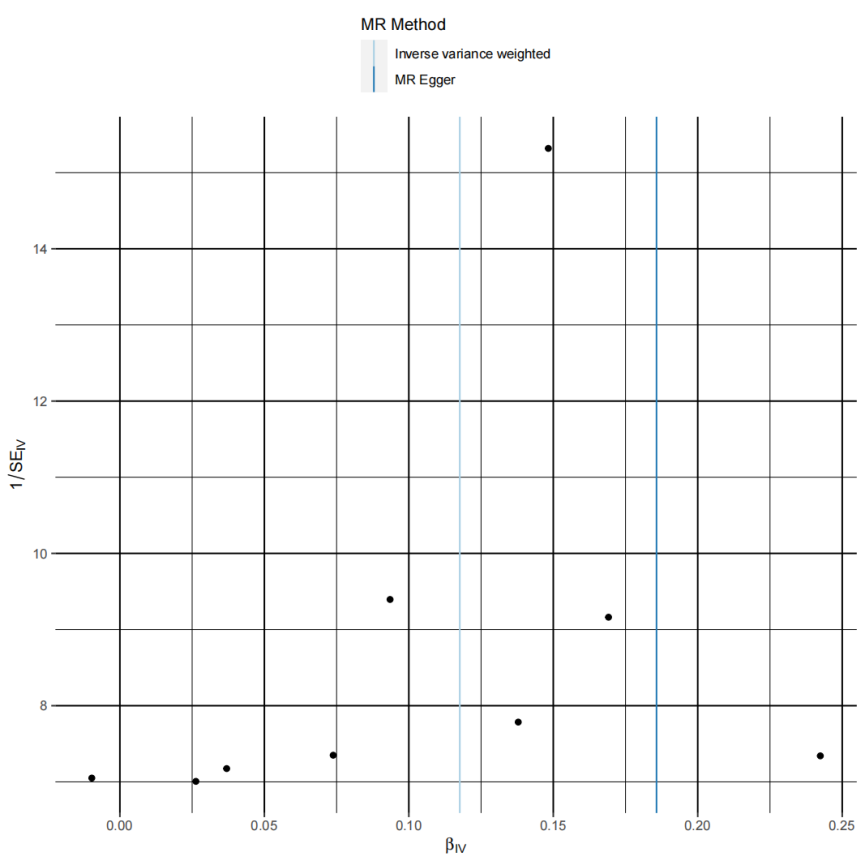


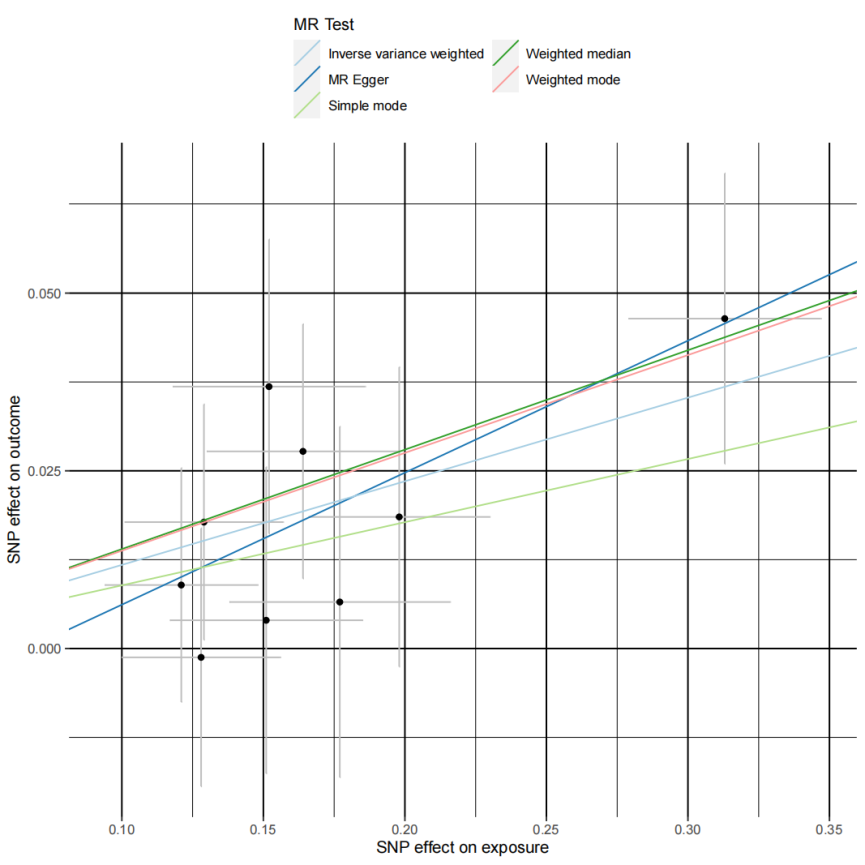


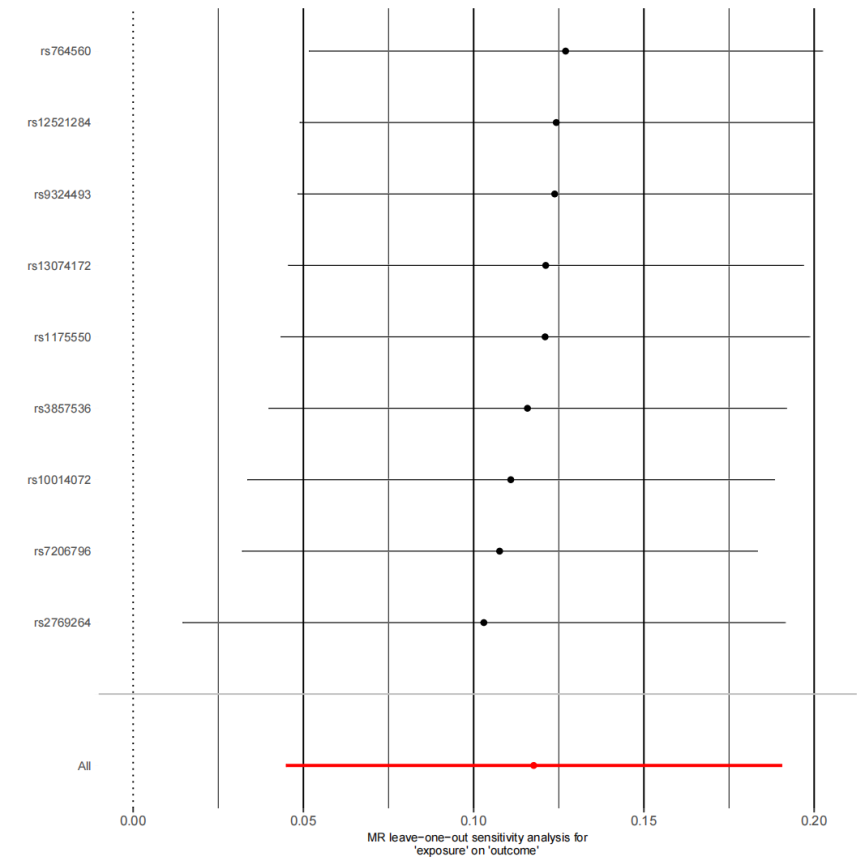


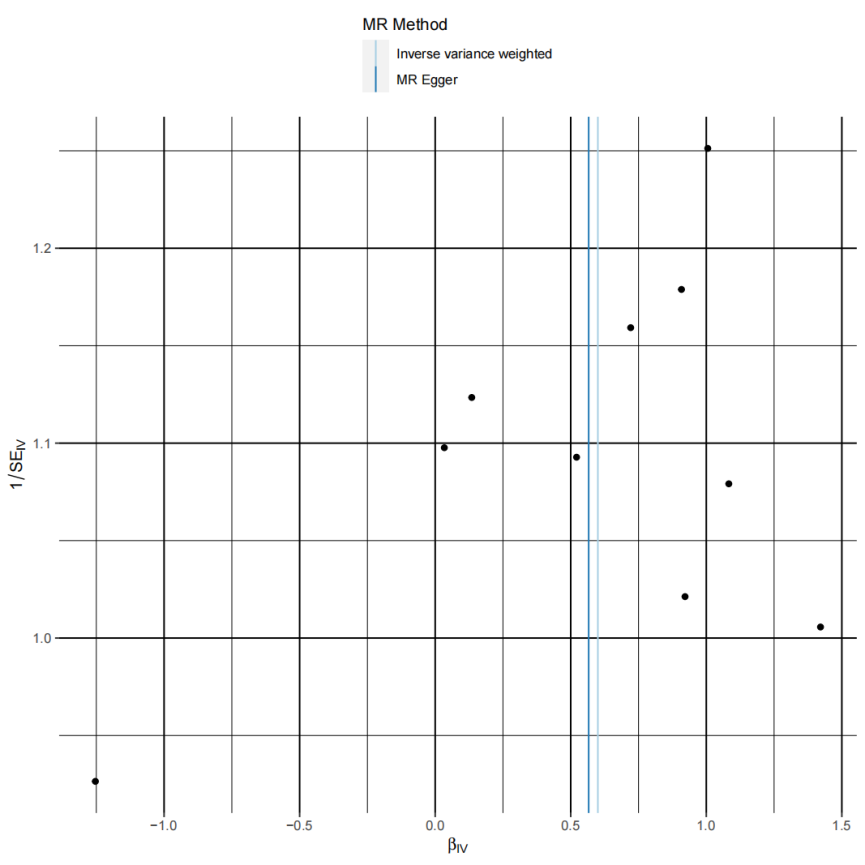


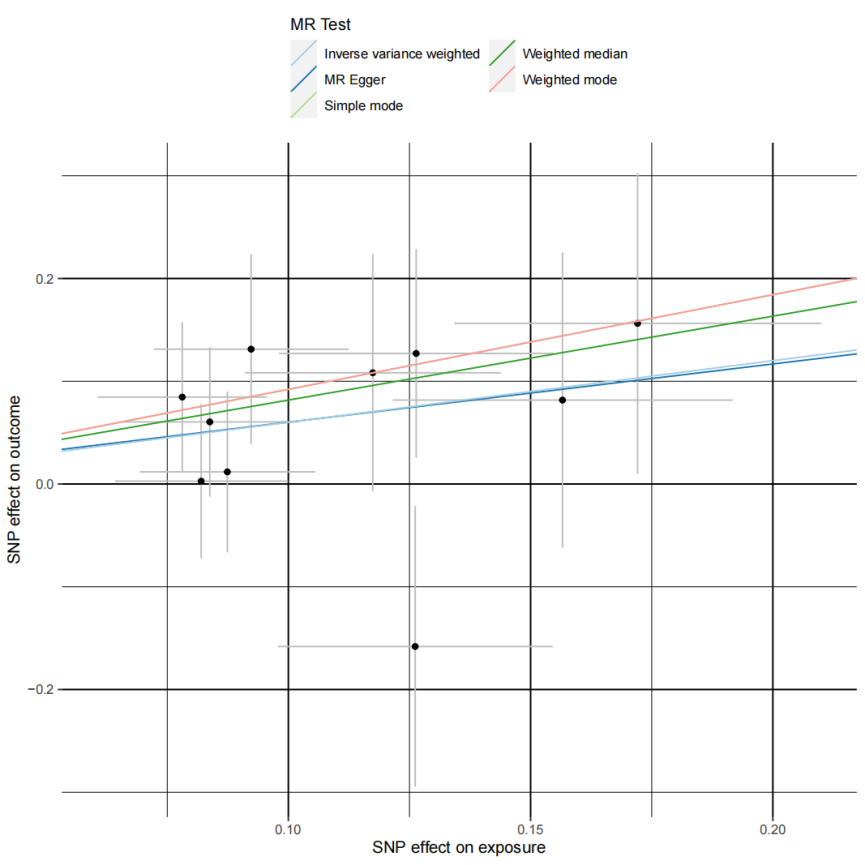


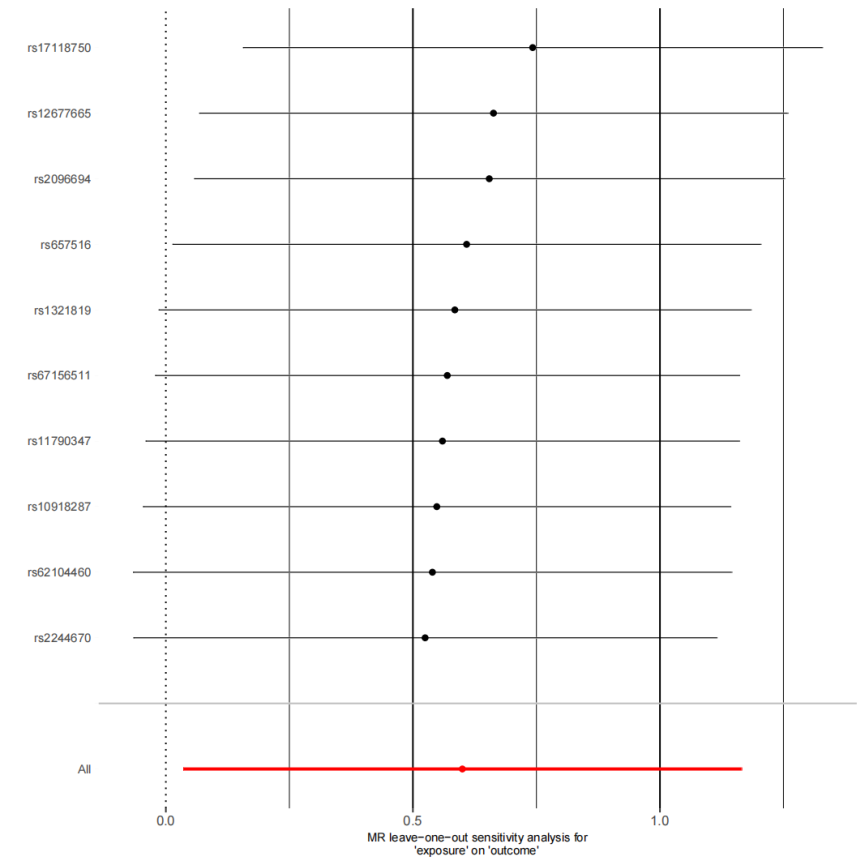

Supplement: Supplementary file 1 — Figure S1: Funnel plot, Scatterplot and Leave‐one‐out test of MR analysis and mediation MR analysis (copper, gut microbiota, and Alzheimer's disease). [file FSN3-13-e70706-s003.docx]

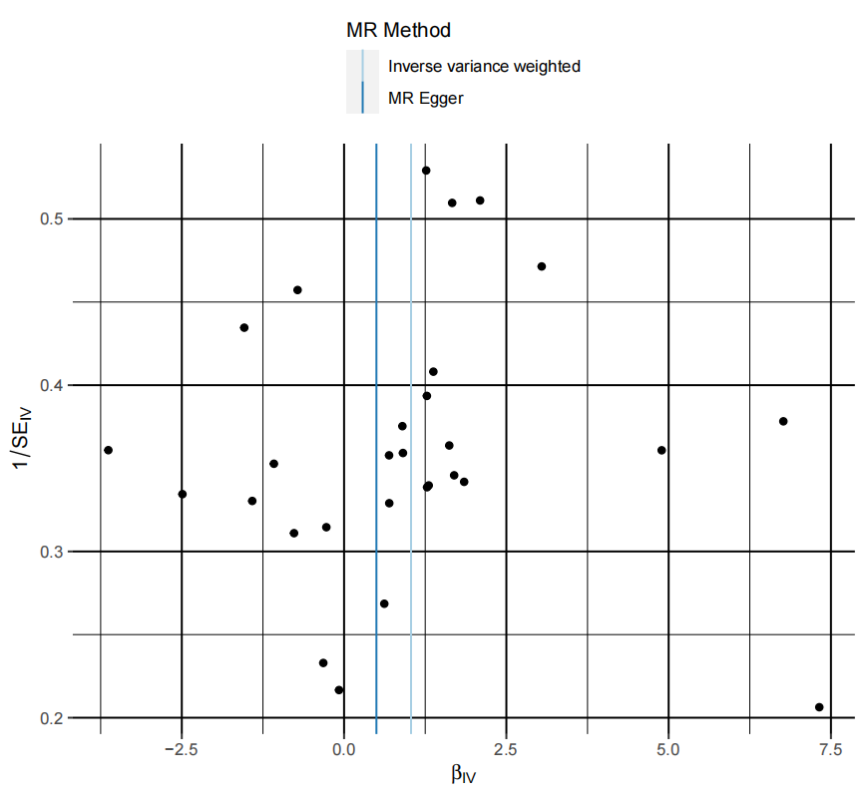


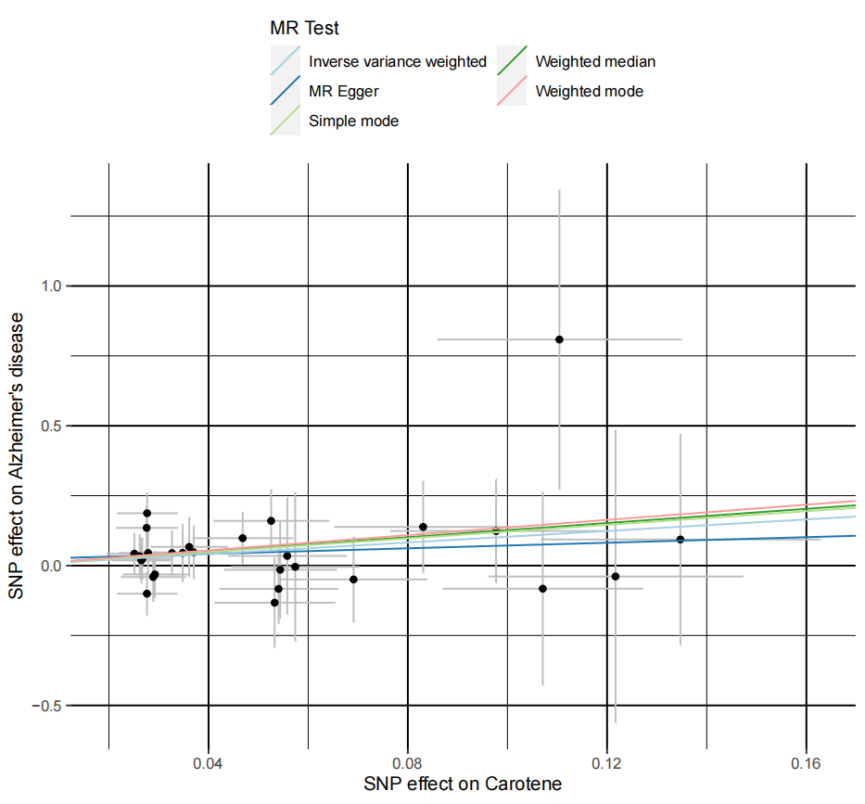


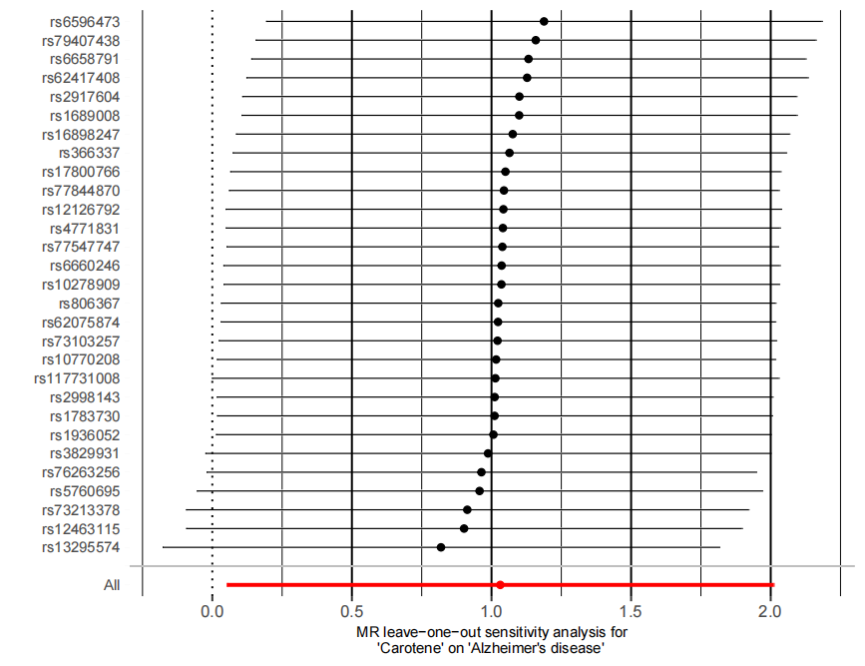


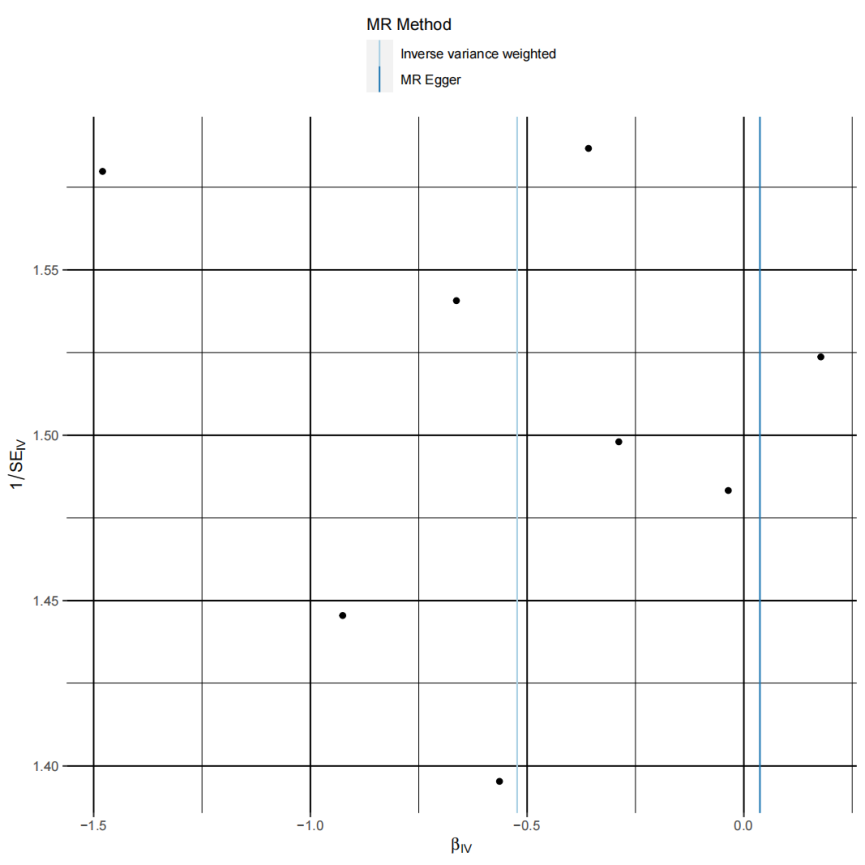


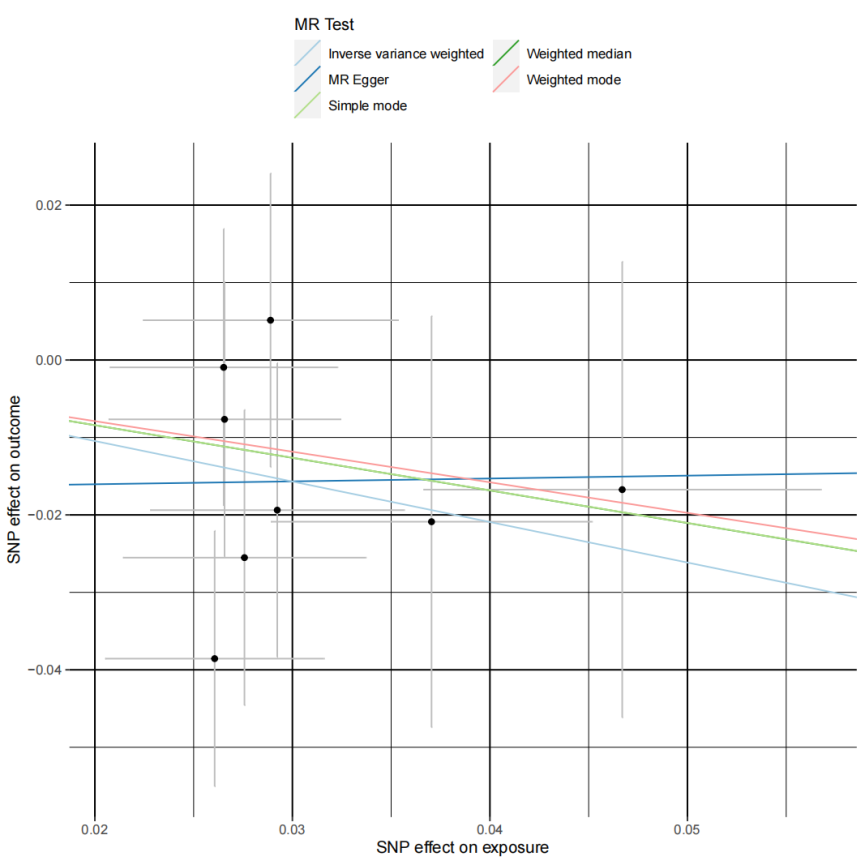


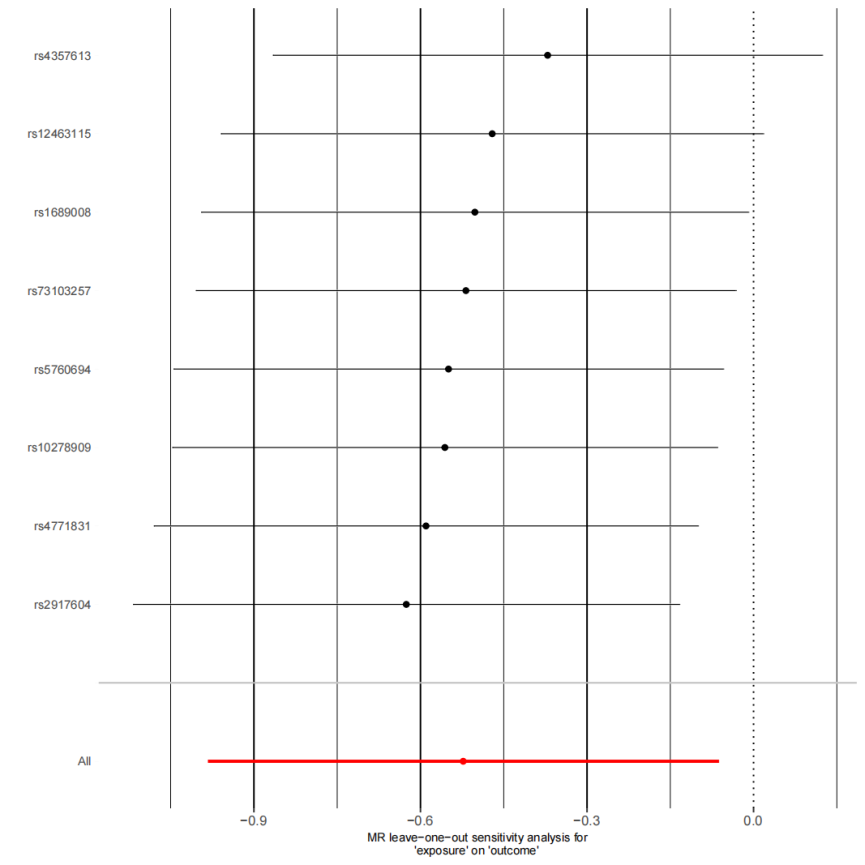


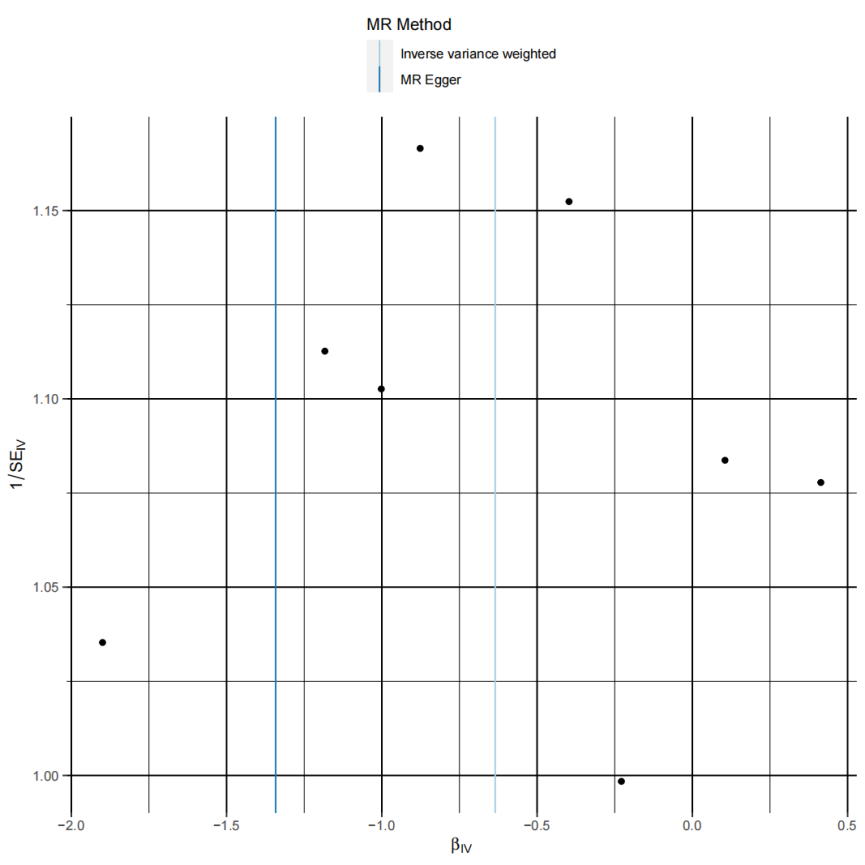


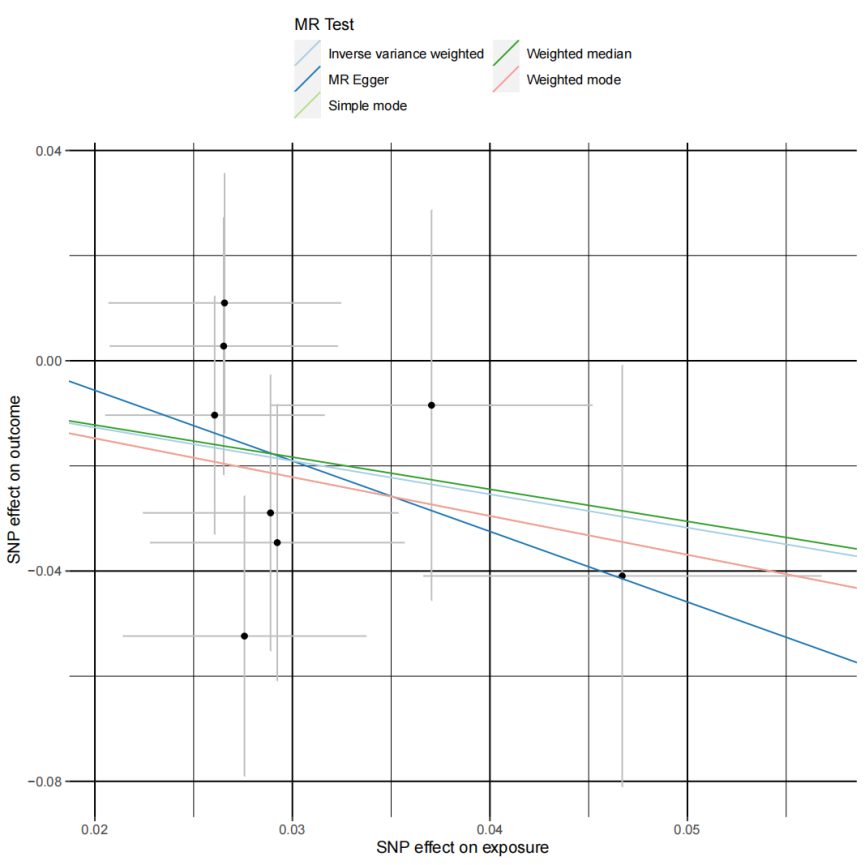


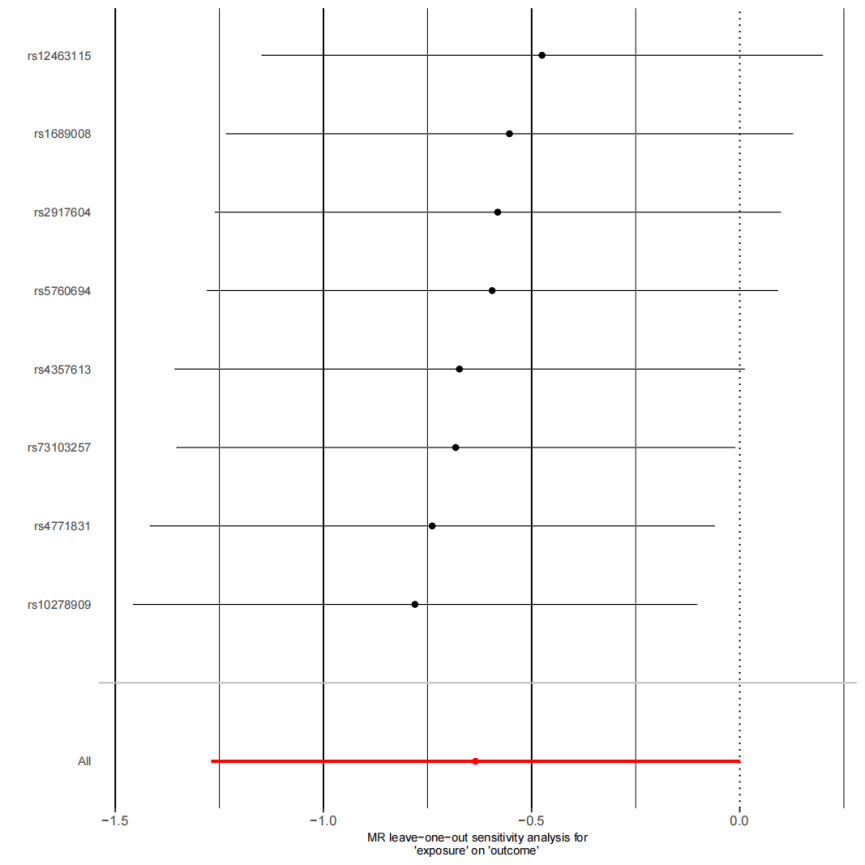


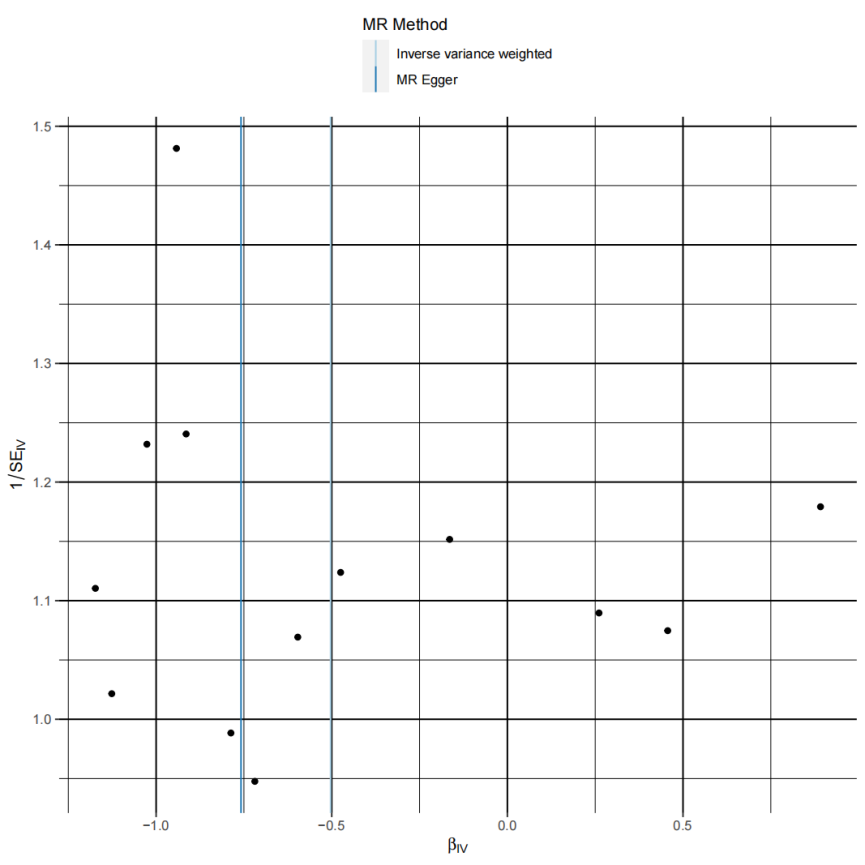


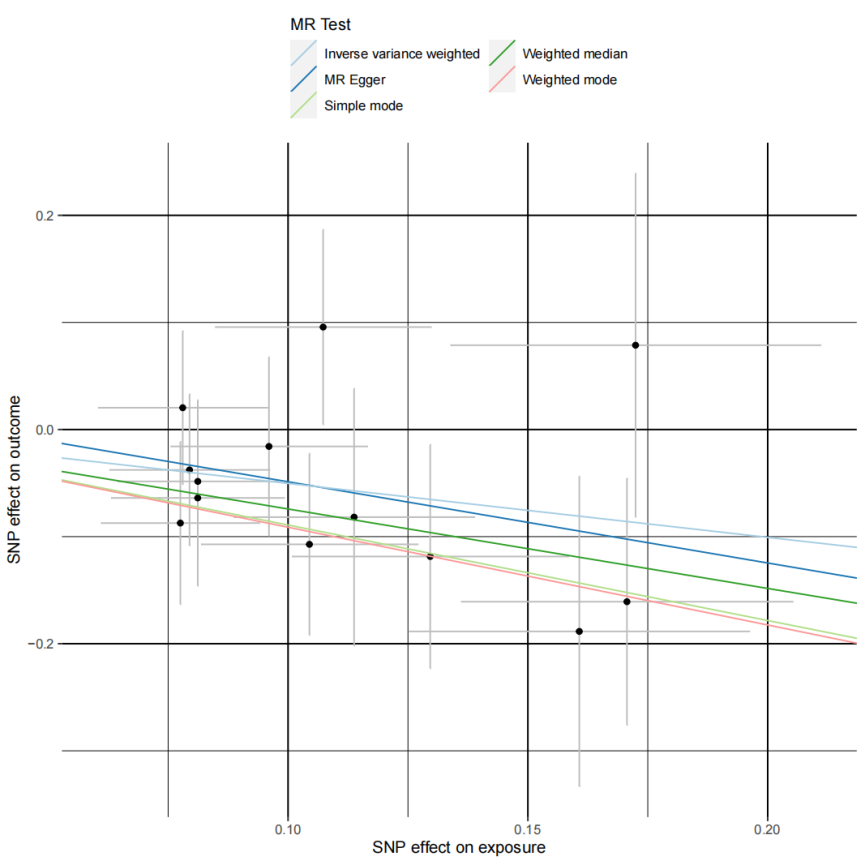


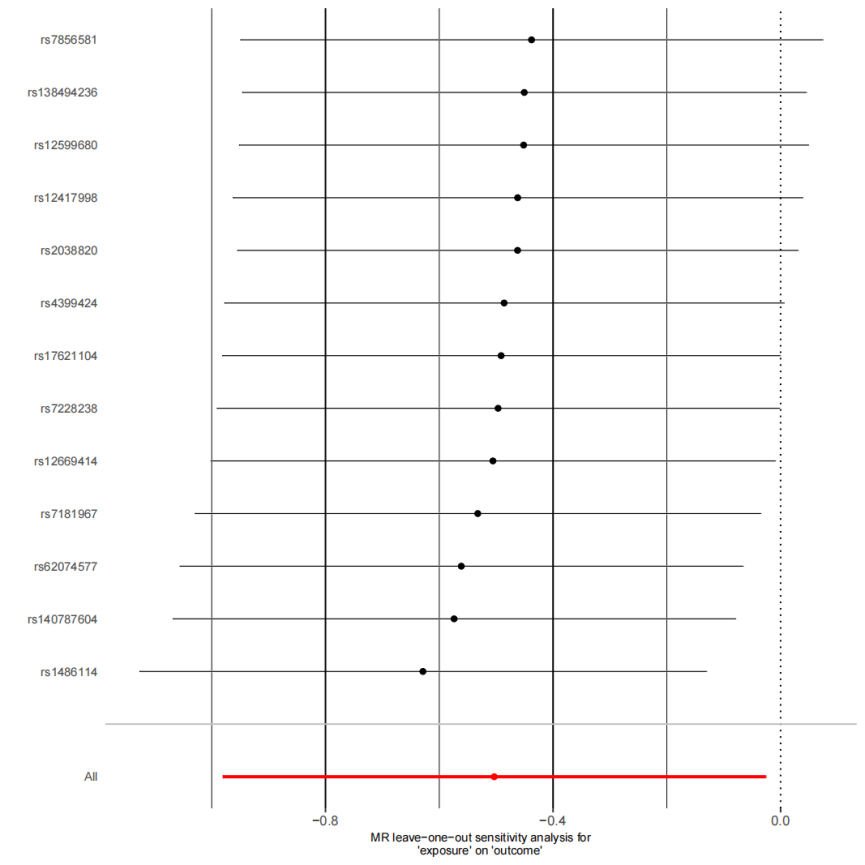


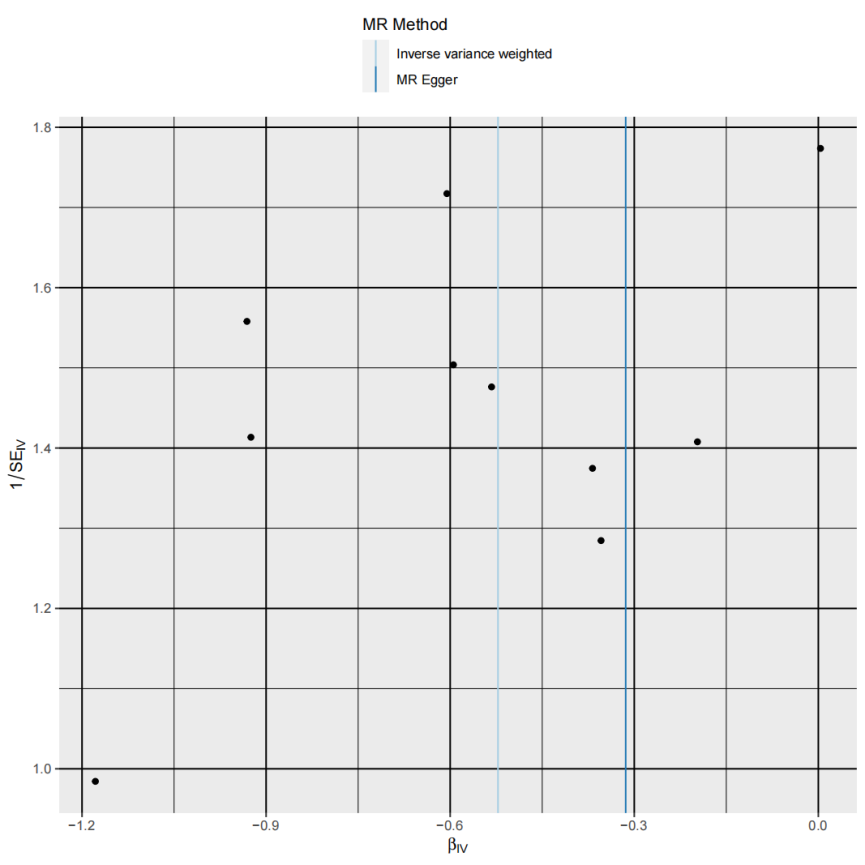


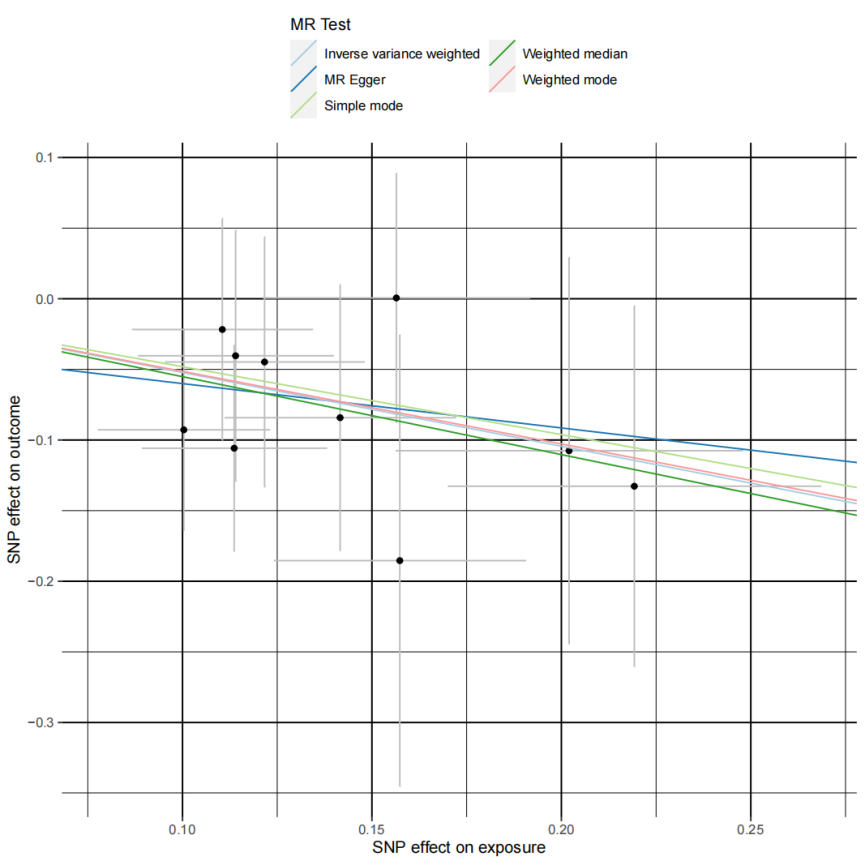


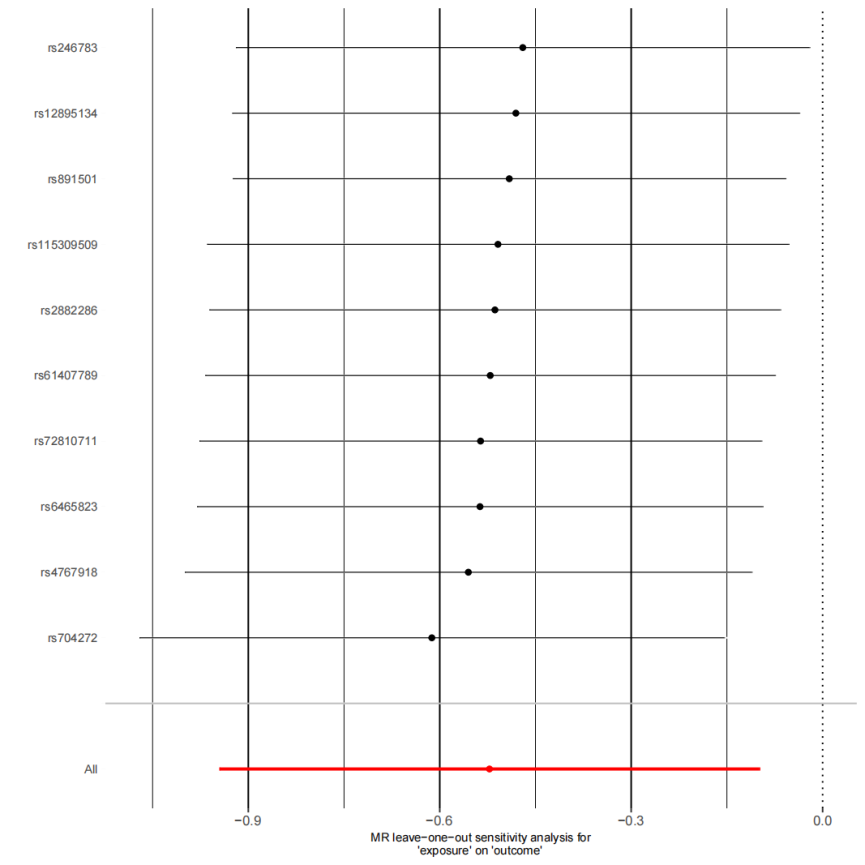

Supplement: Supplementary file 2 — Figure S2: Funnel plot, Scatterplot and Leave‐one‐out test of MR analysis and mediation MR analysis (carotene, gut microbiota, and Alzheimer's disease). [file FSN3-13-e70706-s001.docx]
