# Supplementary material for: Human Trace Elements, Gut Microbiota, and Alzheimer's Disease: Insights From Multistage Mendelian Randomization Analysis
Source: Food Sci Nutr. 2025 Aug 12;13(8):e70706. doi: 10.1002/fsn3.70706 (PMC12340980; doi:10.1002/fsn3.70706)
Supplement: Supplementary file 3 — Table S1: Baseline information table for exposure, mediator and outcome. Table S2: Screening results of single nucleotide polymorphism. Table S3: Mendelian randomization analysis and sensitivity analysis of genetic causality between trace element, gut microbiota and Alzheimer's disease. [file FSN3-13-e70706-s002.docx]

| Table S1. Baseline information table for exposure, mediator and outcome | | | | | |
| --- | --- | --- | --- | --- | --- |
| **Subject** | **Year** | **Study/Author** | **Sample size** | **ID** | **Links for data download** |
| **Exposure** |  |  |  |  |  |
| Iron | 2018 | OpenGWAS | 64,979 | ukb-b-20447 | <https://gwas.mrcieu.ac.uk/datasets/ukb-b-20447/> |
| Sodium | 2019 | OpenGWAS | 127,304 | bbj-a-43 | <https://gwas.mrcieu.ac.uk/datasets/bbj-a-43/> |
| Potassium | 2018 | OpenGWAS | 64,979 | ukb-b-17881 | <https://gwas.mrcieu.ac.uk/datasets/ukb-b-17881/> |
| Zinc | 2013 | OpenGWAS | 2,603 | ieu-a-1079 | <https://gwas.mrcieu.ac.uk/datasets/ieu-a-1079/> |
| Copper | 2013 | OpenGWAS | 2,603 | ieu-a-1073 | <https://gwas.mrcieu.ac.uk/datasets/ieu-a-1073/> |
| Calcium | 2018 | OpenGWAS | 64,979 | ukb-b-8951 | <https://gwas.mrcieu.ac.uk/datasets/ukb-b-8951/> |
| Phosphorus | 2019 | OpenGWAS | 42,793 | bbj-a-45 | <https://gwas.mrcieu.ac.uk/datasets/bbj-a-45/> |
| Selenium | 2013 | OpenGWAS | 2,603 | ieu-a-1077 | <https://gwas.mrcieu.ac.uk/datasets/ieu-a-1077/> |
| Magnesium | 2018 | OpenGWAS | 64,979 | ukb-b-7372 | <https://gwas.mrcieu.ac.uk/datasets/ukb-b-7372/> |
| Vitamin A | 2018 | OpenGWAS | 460,351 | ukb-b-9596 | <https://gwas.mrcieu.ac.uk/datasets/ukb-b-9596/> |
| Vitamin B6 | 2018 | OpenGWAS | 64,979 | ukb-b-7864 | <https://gwas.mrcieu.ac.uk/datasets/ukb-b-7864/> |
| Vitamin B12 | 2018 | OpenGWAS | 64,979 | ukb-b-19524 | <https://gwas.mrcieu.ac.uk/datasets/ukb-b-19524/> |
| Vitamin C | 2018 | OpenGWAS | 64,979 | ukb-b-19390 | <https://gwas.mrcieu.ac.uk/datasets/ukb-b-19390/> |
| Vitamin D | 2018 | OpenGWAS | 64,979 | ukb-b-18593 | <https://gwas.mrcieu.ac.uk/datasets/ukb-b-18593/> |
| Vitamin E | 2018 | OpenGWAS | 64,979 | ukb-b-6888 | <https://gwas.mrcieu.ac.uk/datasets/ukb-b-6888/> |
| Carotene | 2018 | OpenGWAS | 64,979 | ukb-b-16202 | <https://gwas.mrcieu.ac.uk/datasets/ukb-b-16202/> |
| Folate | 2018 | OpenGWAS | 64,979 | ukb-b-11349 | <https://gwas.mrcieu.ac.uk/datasets/ukb-b-11349/> |
| Alzheimer's disease | 2023 | FinnGen consortium | 184,190 | finngen_R10_AD_U_EXMORE | <https://storage.googleapis.com/finngen-public-data-r10/summary_stats/finngen_R10_AD_U_EXMORE.gz> |
| Gut microbiota (412 types) | 2022 | Lopera-Maya EA | 7,738 | PMID: 35115690 | <https://dutchmicrobiomeproject.molgeniscloud.org/> |
| **Mediator** |  |  |  |  |  |
| Gut microbiota (412 types) | 2022 | Lopera-Maya EA | 7,738 | PMID: 35115690 | <https://dutchmicrobiomeproject.molgeniscloud.org/> |
| **Outcome** |  |  |  |  |  |
| Alzheimer's disease | 2023 | FinnGen consortium | 184,190 | finngen_R10_AD_U_EXMORE | <https://storage.googleapis.com/finngen-public-data-r10/summary_stats/finngen_R10_AD_U_EXMORE.gz> |
| Copper | 2013 | OpenGWAS | 2,603 | ieu-a-1073 | <https://gwas.mrcieu.ac.uk/datasets/ieu-a-1073/> |
| Carotene | 2018 | OpenGWAS | 64,979 | ukb-b-16202 | <https://gwas.mrcieu.ac.uk/datasets/ukb-b-16202/> |
| Gut microbiota (412 types) | 2022 | Lopera-Maya EA | 7,738 | PMID: 35115690 | <https://dutchmicrobiomeproject.molgeniscloud.org/> |

| **Table S2.** Screening results of single nucleotide polymorphism | | | | | | | |
| --- | --- | --- | --- | --- | --- | --- | --- |
| **Exposure-Outcome** | **SNP** | **Sample size** | **Beta** | **SE** | **EA** | **NEA** | **F** |
| **Trace element-Alzheimer's disease** |  |  |  |  |  |  |  |
| Iron | rs114738685 | 64,979 | 0.055 | 0.012 | T | C | 22 |
|  | rs116863411 | 64,979 | -0.095 | 0.020 | T | A | 21 |
|  | rs118189684 | 64,979 | 0.059 | 0.012 | C | T | 24 |
|  | rs12884802 | 64,979 | 0.027 | 0.006 | A | G | 20 |
|  | rs1370102 | 64,979 | -0.025 | 0.005 | A | C | 21 |
|  | rs144862520 | 64,979 | -0.068 | 0.015 | T | C | 20 |
|  | rs148244439 | 64,979 | 0.059 | 0.012 | T | C | 23 |
|  | rs155599 | 64,979 | 0.030 | 0.006 | C | T | 26 |
|  | rs17257441 | 64,979 | -0.038 | 0.008 | T | C | 21 |
|  | rs184717452 | 64,979 | -0.042 | 0.009 | C | A | 20 |
|  | rs187093973 | 64,979 | -0.082 | 0.018 | A | G | 20 |
|  | rs1958962 | 64,979 | -0.044 | 0.010 | T | C | 20 |
|  | rs2647238 | 64,979 | -0.025 | 0.005 | C | T | 21 |
|  | rs56256289 | 64,979 | 0.033 | 0.007 | T | C | 22 |
|  | rs60812292 | 64,979 | -0.030 | 0.007 | T | C | 20 |
|  | rs61890331 | 64,979 | -0.075 | 0.017 | T | C | 20 |
|  | rs6463742 | 64,979 | 0.026 | 0.006 | C | T | 22 |
|  | rs71437603 | 64,979 | 0.044 | 0.010 | T | C | 20 |
|  | rs7196805 | 64,979 | 0.036 | 0.008 | T | C | 21 |
|  | rs9297943 | 64,979 | 0.027 | 0.006 | G | T | 22 |
| Sodium | rs10066035 | 127,304 | 0.022 | 0.004 | T | C | 27 |
|  | rs10093809 | 127,304 | 0.030 | 0.006 | G | C | 24 |
|  | rs10143711 | 127,304 | 0.049 | 0.011 | A | G | 21 |
|  | rs10243618 | 127,304 | 0.022 | 0.004 | A | G | 26 |
|  | rs1034660 | 127,304 | 0.020 | 0.004 | C | T | 21 |
|  | rs10898722 | 127,304 | -0.019 | 0.004 | G | T | 20 |
|  | rs11052024 | 127,304 | 0.029 | 0.006 | G | C | 20 |
|  | rs11109811 | 127,304 | -0.038 | 0.006 | G | C | 38 |
|  | rs11191639 | 127,304 | -0.031 | 0.005 | T | C | 38 |
|  | rs113918189 | 127,304 | -0.023 | 0.004 | T | C | 30 |
|  | rs11608804 | 127,304 | -0.034 | 0.007 | A | G | 20 |
|  | rs12037987 | 127,304 | 0.021 | 0.004 | C | T | 22 |
|  | rs12410087 | 127,304 | 0.020 | 0.004 | C | A | 21 |
|  | rs12671313 | 127,304 | -0.019 | 0.004 | A | T | 20 |
|  | rs1275984 | 127,304 | -0.024 | 0.005 | C | A | 24 |
|  | rs13101863 | 127,304 | 0.020 | 0.004 | T | C | 20 |
|  | rs13137656 | 127,304 | 0.020 | 0.004 | T | C | 23 |
|  | rs1347027 | 127,304 | -0.029 | 0.006 | A | G | 24 |
|  | rs147359087 | 127,304 | 0.023 | 0.005 | A | G | 22 |
|  | rs17035646 | 127,304 | 0.019 | 0.004 | A | G | 20 |
|  | rs17446243 | 127,304 | -0.034 | 0.007 | A | G | 25 |
|  | rs2023843 | 127,304 | 0.020 | 0.004 | T | C | 25 |
|  | rs2273640 | 127,304 | 0.032 | 0.004 | A | G | 56 |
|  | rs2764999 | 127,304 | -0.020 | 0.004 | T | C | 24 |
|  | rs2995762 | 127,304 | -0.018 | 0.004 | T | C | 21 |
|  | rs34562254 | 127,304 | -0.025 | 0.004 | A | G | 38 |
|  | rs35397826 | 127,304 | 0.031 | 0.005 | G | A | 33 |
|  | rs35487869 | 127,304 | -0.028 | 0.004 | A | C | 40 |
|  | rs3858704 | 127,304 | -0.024 | 0.004 | G | A | 33 |
|  | rs39999 | 127,304 | -0.046 | 0.006 | G | C | 66 |
|  | rs4664048 | 127,304 | -0.045 | 0.007 | T | A | 45 |
|  | rs474490 | 127,304 | 0.025 | 0.005 | G | T | 27 |
|  | rs4767019 | 127,304 | -0.019 | 0.004 | A | G | 23 |
|  | rs4856203 | 127,304 | -0.031 | 0.007 | C | T | 21 |
|  | rs4919683 | 127,304 | -0.031 | 0.004 | A | C | 61 |
|  | rs58476720 | 127,304 | 0.029 | 0.006 | A | G | 22 |
|  | rs6120974 | 127,304 | -0.031 | 0.007 | C | T | 20 |
|  | rs668174 | 127,304 | -0.021 | 0.004 | C | T | 23 |
|  | rs6810761 | 127,304 | 0.026 | 0.006 | A | G | 20 |
|  | rs6874626 | 127,304 | -0.020 | 0.004 | A | G | 27 |
|  | rs7113624 | 127,304 | -0.035 | 0.005 | A | G | 47 |
|  | rs7215555 | 127,304 | 0.021 | 0.005 | A | G | 22 |
|  | rs7538978 | 127,304 | -0.029 | 0.004 | A | G | 48 |
|  | rs7557395 | 127,304 | -0.036 | 0.005 | G | A | 52 |
|  | rs7772128 | 127,304 | 0.022 | 0.004 | G | A | 24 |
|  | rs77750312 | 127,304 | 0.023 | 0.004 | T | G | 26 |
|  | rs778821 | 127,304 | -0.025 | 0.005 | C | T | 29 |
|  | rs7804041 | 127,304 | -0.019 | 0.004 | T | C | 21 |
|  | rs7828172 | 127,304 | -0.021 | 0.005 | G | A | 21 |
|  | rs807191 | 127,304 | 0.021 | 0.005 | C | A | 22 |
|  | rs9368226 | 127,304 | -0.021 | 0.004 | C | T | 29 |
|  | rs9373914 | 127,304 | 0.018 | 0.004 | A | T | 20 |
|  | rs9440302 | 127,304 | 0.019 | 0.004 | G | A | 20 |
|  | rs9945319 | 127,304 | 0.026 | 0.006 | A | C | 20 |
| Potassium | rs10001929 | 64,979 | -0.035 | 0.008 | A | G | 20 |
|  | rs10764330 | 64,979 | 0.028 | 0.006 | G | A | 22 |
|  | rs10787760 | 64,979 | -0.028 | 0.006 | A | G | 20 |
|  | rs114300683 | 64,979 | -0.115 | 0.025 | A | G | 21 |
|  | rs116028267 | 64,979 | -0.065 | 0.015 | G | C | 20 |
|  | rs11650824 | 64,979 | 0.071 | 0.016 | A | T | 20 |
|  | rs11955242 | 64,979 | 0.029 | 0.006 | G | A | 20 |
|  | rs12296227 | 64,979 | 0.028 | 0.006 | T | G | 21 |
|  | rs12412051 | 64,979 | 0.073 | 0.015 | C | G | 24 |
|  | rs12471085 | 64,979 | 0.051 | 0.011 | T | C | 20 |
|  | rs13313376 | 64,979 | -0.035 | 0.008 | T | A | 21 |
|  | rs145857065 | 64,979 | -0.083 | 0.017 | C | T | 23 |
|  | rs148244439 | 64,979 | 0.057 | 0.012 | T | C | 21 |
|  | rs2745938 | 64,979 | 0.026 | 0.006 | T | G | 21 |
|  | rs311678 | 64,979 | -0.029 | 0.006 | T | C | 20 |
|  | rs34280758 | 64,979 | 0.049 | 0.011 | T | C | 20 |
|  | rs34751238 | 64,979 | 0.030 | 0.007 | A | G | 21 |
|  | rs35579431 | 64,979 | 0.029 | 0.006 | T | C | 23 |
|  | rs361294 | 64,979 | -0.028 | 0.006 | C | A | 23 |
|  | rs3772928 | 64,979 | -0.027 | 0.006 | C | T | 23 |
|  | rs589835 | 64,979 | -0.031 | 0.007 | A | G | 20 |
|  | rs62431610 | 64,979 | 0.038 | 0.009 | C | T | 20 |
|  | rs62438576 | 64,979 | 0.051 | 0.011 | C | T | 20 |
|  | rs633683 | 64,979 | 0.025 | 0.006 | C | T | 20 |
|  | rs7040926 | 64,979 | -0.051 | 0.011 | C | T | 21 |
|  | rs7296784 | 64,979 | -0.026 | 0.006 | G | A | 20 |
|  | rs7479680 | 64,979 | -0.038 | 0.008 | C | A | 24 |
|  | rs77126457 | 64,979 | 0.105 | 0.023 | A | G | 21 |
|  | rs77824658 | 64,979 | 0.044 | 0.010 | G | A | 21 |
|  | rs7816339 | 64,979 | 0.025 | 0.006 | T | G | 20 |
|  | rs79124792 | 64,979 | -0.048 | 0.011 | T | C | 20 |
| Zinc | rs10484100 | 2,603 | -0.209 | 0.045 | G | A | 22 |
|  | rs10931753 | 2,603 | -0.129 | 0.028 | C | G | 21 |
|  | rs11232535 | 2,603 | 0.325 | 0.065 | C | T | 25 |
|  | rs11763353 | 2,603 | -0.192 | 0.039 | G | A | 24 |
|  | rs1532423 | 2,603 | -0.178 | 0.026 | G | A | 47 |
|  | rs17097781 | 2,603 | -0.242 | 0.055 | T | C | 19 |
|  | rs17511001 | 2,603 | 0.132 | 0.029 | A | G | 21 |
|  | rs2120019 | 2,603 | -0.287 | 0.033 | C | T | 76 |
|  | rs4333127 | 2,603 | 0.218 | 0.047 | A | G | 22 |
|  | rs6545343 | 2,603 | 0.232 | 0.052 | G | C | 20 |
|  | rs7148590 | 2,603 | -0.140 | 0.026 | A | G | 29 |
|  | rs7569234 | 2,603 | 0.539 | 0.118 | C | T | 21 |
|  | rs8099461 | 2,603 | -0.278 | 0.061 | G | A | 21 |
| Copper | rs10014072 | 2,603 | -0.164 | 0.034 | G | A | 23 |
|  | rs1175550 | 2,603 | 0.198 | 0.032 | G | A | 38 |
|  | rs12153606 | 2,603 | -0.159 | 0.034 | T | G | 22 |
|  | rs12582659 | 2,603 | 1.262 | 0.270 | C | T | 22 |
|  | rs13074172 | 2,603 | 0.121 | 0.027 | G | A | 20 |
|  | rs2769264 | 2,603 | 0.313 | 0.034 | G | T | 85 |
|  | rs3857536 | 2,603 | -0.129 | 0.028 | T | C | 21 |
|  | rs572585 | 2,603 | -0.137 | 0.031 | C | T | 20 |
|  | rs7206796 | 2,603 | -0.152 | 0.034 | T | C | 20 |
|  | rs764560 | 2,603 | -0.128 | 0.028 | T | C | 21 |
|  | rs9324493 | 2,603 | -0.177 | 0.039 | G | A | 21 |
| Calcium | rs11030416 | 64,979 | -0.065 | 0.014 | A | G | 23 |
|  | rs11088797 | 64,979 | 0.040 | 0.008 | A | G | 22 |
|  | rs115490327 | 64,979 | -0.132 | 0.030 | A | C | 20 |
|  | rs117456360 | 64,979 | 0.063 | 0.014 | A | G | 21 |
|  | rs11931892 | 64,979 | 0.032 | 0.007 | C | A | 20 |
|  | rs1219820 | 64,979 | -0.076 | 0.016 | T | C | 23 |
|  | rs12618785 | 64,979 | 0.027 | 0.006 | G | C | 24 |
|  | rs12761198 | 64,979 | 0.047 | 0.010 | C | T | 20 |
|  | rs13425632 | 64,979 | -0.032 | 0.007 | A | G | 20 |
|  | rs142996120 | 64,979 | 0.088 | 0.019 | G | A | 20 |
|  | rs1714800 | 64,979 | 0.028 | 0.006 | C | G | 24 |
|  | rs17712285 | 64,979 | -0.071 | 0.015 | G | A | 23 |
|  | rs1974821 | 64,979 | 0.036 | 0.008 | A | G | 22 |
|  | rs2443773 | 64,979 | 0.027 | 0.005 | G | T | 25 |
|  | rs35683760 | 64,979 | -0.031 | 0.006 | G | A | 23 |
|  | rs39308 | 64,979 | 0.030 | 0.006 | A | G | 23 |
|  | rs4535437 | 64,979 | 0.032 | 0.006 | G | A | 25 |
|  | rs4988235 | 64,979 | 0.032 | 0.006 | A | G | 29 |
|  | rs61847291 | 64,979 | -0.027 | 0.006 | C | T | 20 |
|  | rs62347998 | 64,979 | 0.064 | 0.014 | T | C | 22 |
|  | rs73238581 | 64,979 | -0.027 | 0.006 | A | C | 23 |
|  | rs74582077 | 64,979 | -0.056 | 0.013 | C | G | 20 |
|  | rs7464794 | 64,979 | -0.026 | 0.005 | C | T | 24 |
|  | rs753899 | 64,979 | 0.046 | 0.009 | C | T | 25 |
|  | rs77126457 | 64,979 | 0.104 | 0.023 | A | G | 20 |
|  | rs8067154 | 64,979 | -0.030 | 0.007 | A | G | 21 |
|  | rs8109178 | 64,979 | 0.039 | 0.008 | T | A | 22 |
| Phosphorus | rs10743976 | 42,793 | 0.044 | 0.009 | C | A | 24 |
|  | rs10913906 | 42,793 | 0.045 | 0.010 | G | A | 20 |
|  | rs1106357 | 42,793 | 0.063 | 0.007 | T | C | 74 |
|  | rs12368351 | 42,793 | -0.072 | 0.008 | A | G | 76 |
|  | rs13385955 | 42,793 | -0.045 | 0.010 | T | C | 22 |
|  | rs1453382 | 42,793 | -0.040 | 0.009 | T | A | 20 |
|  | rs16915737 | 42,793 | -0.036 | 0.007 | T | C | 24 |
|  | rs17060705 | 42,793 | 0.056 | 0.010 | A | G | 32 |
|  | rs2732179 | 42,793 | -0.033 | 0.007 | C | G | 21 |
|  | rs35186465 | 42,793 | 0.045 | 0.008 | A | G | 33 |
|  | rs35716097 | 42,793 | -0.073 | 0.007 | T | C | 99 |
|  | rs4243177 | 42,793 | 0.031 | 0.007 | G | A | 21 |
|  | rs4654960 | 42,793 | 0.060 | 0.009 | G | T | 47 |
|  | rs56195122 | 42,793 | -0.063 | 0.014 | A | G | 20 |
|  | rs6484900 | 42,793 | 0.036 | 0.008 | T | A | 20 |
|  | rs73743323 | 42,793 | -0.141 | 0.019 | T | C | 54 |
| Selenium | rs10023369 | 2,603 | -0.144 | 0.029 | A | G | 25 |
|  | rs11114989 | 2,603 | 0.130 | 0.029 | A | G | 20 |
|  | rs11779526 | 2,603 | 0.153 | 0.032 | T | A | 23 |
|  | rs1492483 | 2,603 | 0.130 | 0.029 | G | A | 20 |
|  | rs2461362 | 2,603 | -0.242 | 0.054 | T | C | 20 |
|  | rs3785832 | 2,603 | -0.146 | 0.031 | C | T | 22 |
|  | rs4779561 | 2,603 | -0.139 | 0.031 | T | C | 20 |
|  | rs4950779 | 2,603 | 0.673 | 0.132 | C | T | 26 |
|  | rs566108 | 2,603 | 0.201 | 0.045 | C | A | 20 |
|  | rs6863754 | 2,603 | -1.422 | 0.314 | A | C | 21 |
|  | rs713550 | 2,603 | -0.251 | 0.056 | T | G | 20 |
|  | rs7163368 | 2,603 | 0.158 | 0.034 | C | T | 22 |
|  | rs7700970 | 2,603 | 0.265 | 0.037 | T | C | 51 |
|  | rs9309294 | 2,603 | -0.133 | 0.030 | G | A | 20 |
| Magnesium | rs111419911 | 64,979 | -0.029 | 0.006 | G | A | 25 |
|  | rs111893801 | 64,979 | -0.053 | 0.012 | T | C | 21 |
|  | rs114283943 | 64,979 | 0.055 | 0.012 | G | A | 20 |
|  | rs114575778 | 64,979 | -0.145 | 0.027 | A | G | 28 |
|  | rs114905478 | 64,979 | -0.078 | 0.017 | A | G | 20 |
|  | rs114989460 | 64,979 | 0.096 | 0.021 | C | T | 21 |
|  | rs116028267 | 64,979 | -0.067 | 0.014 | G | C | 21 |
|  | rs11650824 | 64,979 | 0.070 | 0.016 | A | T | 20 |
|  | rs116740989 | 64,979 | 0.118 | 0.025 | A | C | 22 |
|  | rs116979507 | 64,979 | 0.064 | 0.014 | T | C | 21 |
|  | rs11961853 | 64,979 | -0.032 | 0.007 | T | C | 20 |
|  | rs12268032 | 64,979 | -0.031 | 0.007 | A | G | 20 |
|  | rs1247081 | 64,979 | 0.026 | 0.005 | T | G | 23 |
|  | rs13123116 | 64,979 | 0.082 | 0.018 | A | T | 20 |
|  | rs140172022 | 64,979 | -0.125 | 0.028 | G | A | 20 |
|  | rs144862520 | 64,979 | -0.070 | 0.015 | T | C | 21 |
|  | rs147150587 | 64,979 | 0.128 | 0.028 | A | G | 22 |
|  | rs1559583 | 64,979 | -0.030 | 0.007 | C | T | 22 |
|  | rs2034876 | 64,979 | -0.048 | 0.011 | G | A | 20 |
|  | rs2745938 | 64,979 | 0.027 | 0.006 | T | G | 23 |
|  | rs2807980 | 64,979 | 0.027 | 0.006 | G | A | 21 |
|  | rs2826224 | 64,979 | 0.030 | 0.007 | C | T | 20 |
|  | rs4535437 | 64,979 | 0.030 | 0.006 | G | A | 24 |
|  | rs573905 | 64,979 | 0.028 | 0.005 | G | A | 26 |
|  | rs62290893 | 64,979 | -0.029 | 0.006 | T | C | 21 |
|  | rs7022555 | 64,979 | -0.121 | 0.025 | T | C | 23 |
|  | rs72712347 | 64,979 | -0.058 | 0.013 | A | C | 20 |
|  | rs72816351 | 64,979 | 0.043 | 0.009 | G | C | 20 |
|  | rs7339029 | 64,979 | 0.036 | 0.008 | T | G | 21 |
|  | rs7542974 | 64,979 | 0.027 | 0.006 | A | G | 20 |
|  | rs76330086 | 64,979 | -0.102 | 0.022 | T | C | 21 |
|  | rs77126457 | 64,979 | 0.115 | 0.023 | A | G | 25 |
| Vitamin A | rs10248388 | 460,351 | 0.003 | 0.001 | C | A | 22 |
|  | rs112549610 | 460,351 | -0.002 | 0.000 | A | C | 20 |
|  | rs117156918 | 460,351 | -0.002 | 0.000 | C | G | 20 |
|  | rs12213562 | 460,351 | 0.002 | 0.000 | T | C | 20 |
|  | rs13131030 | 460,351 | -0.002 | 0.000 | A | G | 20 |
|  | rs1478684 | 460,351 | 0.002 | 0.000 | G | T | 23 |
|  | rs1953042 | 460,351 | 0.002 | 0.000 | C | T | 22 |
|  | rs2581667 | 460,351 | 0.002 | 0.000 | G | A | 22 |
|  | rs4131899 | 460,351 | -0.001 | 0.000 | T | C | 23 |
|  | rs4379489 | 460,351 | 0.002 | 0.000 | G | A | 21 |
|  | rs6005584 | 460,351 | -0.002 | 0.001 | C | G | 21 |
|  | rs62189516 | 460,351 | 0.002 | 0.001 | C | T | 20 |
|  | rs72774943 | 460,351 | -0.003 | 0.001 | T | C | 21 |
|  | rs72833036 | 460,351 | 0.003 | 0.001 | G | A | 31 |
|  | rs7330997 | 460,351 | -0.002 | 0.000 | C | T | 23 |
|  | rs78367736 | 460,351 | 0.003 | 0.001 | T | C | 20 |
|  | rs9982066 | 460,351 | 0.001 | 0.000 | A | C | 25 |
| Vitamin B6 | rs10132524 | 64,979 | -0.034 | 0.008 | G | A | 20 |
|  | rs10138490 | 64,979 | -0.053 | 0.011 | C | T | 22 |
|  | rs12198456 | 64,979 | 0.092 | 0.020 | T | C | 22 |
|  | rs12226112 | 64,979 | 0.028 | 0.006 | T | G | 24 |
|  | rs12296227 | 64,979 | 0.027 | 0.006 | T | G | 20 |
|  | rs12412051 | 64,979 | 0.071 | 0.015 | C | G | 22 |
|  | rs12521185 | 64,979 | 0.033 | 0.007 | A | G | 20 |
|  | rs13002787 | 64,979 | -0.053 | 0.012 | T | A | 20 |
|  | rs141933624 | 64,979 | -0.090 | 0.019 | A | G | 22 |
|  | rs145857065 | 64,979 | -0.078 | 0.017 | C | T | 20 |
|  | rs155599 | 64,979 | 0.034 | 0.006 | C | T | 33 |
|  | rs183178622 | 64,979 | -0.099 | 0.021 | T | C | 23 |
|  | rs188211816 | 64,979 | -0.079 | 0.016 | A | G | 23 |
|  | rs2280351 | 64,979 | 0.026 | 0.006 | A | G | 20 |
|  | rs2727550 | 64,979 | 0.026 | 0.006 | A | G | 20 |
|  | rs34938615 | 64,979 | -0.122 | 0.026 | G | A | 21 |
|  | rs361294 | 64,979 | -0.027 | 0.006 | C | A | 21 |
|  | rs3745438 | 64,979 | -0.071 | 0.016 | C | T | 21 |
|  | rs3772928 | 64,979 | -0.029 | 0.006 | C | T | 28 |
|  | rs385441 | 64,979 | 0.026 | 0.006 | T | G | 20 |
|  | rs59570585 | 64,979 | 0.106 | 0.023 | A | C | 21 |
|  | rs61890331 | 64,979 | -0.076 | 0.017 | T | C | 20 |
|  | rs67450584 | 64,979 | 0.037 | 0.007 | T | C | 24 |
|  | rs7205927 | 64,979 | -0.026 | 0.006 | C | A | 22 |
|  | rs73181599 | 64,979 | 0.127 | 0.028 | A | G | 20 |
|  | rs74640671 | 64,979 | -0.127 | 0.027 | T | C | 22 |
|  | rs77008857 | 64,979 | -0.068 | 0.015 | C | A | 21 |
|  | rs77806858 | 64,979 | -0.051 | 0.011 | C | T | 23 |
|  | rs78091867 | 64,979 | 0.087 | 0.020 | C | G | 20 |
|  | rs9560457 | 64,979 | 0.026 | 0.006 | T | C | 21 |
| Vitamin B12 | rs10924919 | 64,979 | -0.029 | 0.006 | T | C | 26 |
|  | rs112961770 | 64,979 | -0.089 | 0.018 | C | G | 23 |
|  | rs114250802 | 64,979 | 0.086 | 0.019 | G | A | 20 |
|  | rs117428413 | 64,979 | -0.066 | 0.015 | A | C | 20 |
|  | rs12776611 | 64,979 | -0.088 | 0.019 | A | G | 22 |
|  | rs12943383 | 64,979 | 0.028 | 0.006 | A | G | 21 |
|  | rs12944377 | 64,979 | -0.025 | 0.006 | C | T | 20 |
|  | rs13100032 | 64,979 | 0.044 | 0.010 | C | G | 21 |
|  | rs139450442 | 64,979 | -0.079 | 0.018 | G | A | 20 |
|  | rs1419875 | 64,979 | -0.032 | 0.007 | G | T | 21 |
|  | rs142650015 | 64,979 | -0.125 | 0.028 | A | C | 20 |
|  | rs148901823 | 64,979 | -0.049 | 0.010 | G | A | 23 |
|  | rs1546109 | 64,979 | 0.025 | 0.006 | C | G | 20 |
|  | rs2262245 | 64,979 | -0.026 | 0.006 | C | T | 20 |
|  | rs2749504 | 64,979 | -0.026 | 0.006 | A | G | 20 |
|  | rs35521019 | 64,979 | -0.025 | 0.006 | T | C | 20 |
|  | rs388561 | 64,979 | 0.040 | 0.009 | C | T | 22 |
|  | rs4147823 | 64,979 | -0.030 | 0.007 | C | A | 20 |
|  | rs4717478 | 64,979 | -0.029 | 0.006 | A | G | 20 |
|  | rs4880772 | 64,979 | 0.066 | 0.015 | G | A | 20 |
|  | rs58801062 | 64,979 | -0.036 | 0.008 | G | T | 20 |
|  | rs61504980 | 64,979 | 0.029 | 0.006 | A | G | 20 |
|  | rs61994378 | 64,979 | 0.093 | 0.020 | C | T | 22 |
|  | rs67568068 | 64,979 | -0.032 | 0.007 | C | T | 23 |
|  | rs9819211 | 64,979 | 0.031 | 0.007 | A | C | 21 |
| Vitamin C | rs114158296 | 64,979 | 0.127 | 0.029 | T | C | 20 |
|  | rs114598078 | 64,979 | 0.066 | 0.014 | T | C | 23 |
|  | rs11650824 | 64,979 | 0.079 | 0.016 | A | T | 25 |
|  | rs117381692 | 64,979 | -0.066 | 0.015 | A | G | 20 |
|  | rs11986565 | 64,979 | 0.026 | 0.006 | G | T | 21 |
|  | rs12104154 | 64,979 | -0.050 | 0.011 | T | C | 21 |
|  | rs140617399 | 64,979 | 0.118 | 0.026 | G | T | 21 |
|  | rs17482258 | 64,979 | 0.043 | 0.009 | T | C | 21 |
|  | rs1883993 | 64,979 | 0.045 | 0.009 | A | G | 23 |
|  | rs2018201 | 64,979 | -0.081 | 0.017 | G | T | 22 |
|  | rs2485652 | 64,979 | 0.025 | 0.006 | G | A | 20 |
|  | rs4238567 | 64,979 | 0.025 | 0.006 | C | T | 21 |
|  | rs4481190 | 64,979 | -0.031 | 0.006 | C | A | 28 |
|  | rs56161640 | 64,979 | -0.034 | 0.007 | T | A | 21 |
|  | rs61868302 | 64,979 | -0.057 | 0.012 | T | C | 23 |
|  | rs6812943 | 64,979 | 0.025 | 0.006 | A | G | 20 |
|  | rs7626478 | 64,979 | 0.028 | 0.006 | A | G | 21 |
|  | rs78506130 | 64,979 | 0.050 | 0.011 | G | A | 20 |
|  | rs79456937 | 64,979 | 0.057 | 0.013 | T | C | 21 |
|  | rs79757038 | 64,979 | -0.087 | 0.020 | T | C | 20 |
|  | rs913833 | 64,979 | -0.048 | 0.011 | G | A | 20 |
|  | rs9540734 | 64,979 | -0.026 | 0.005 | A | G | 22 |
| Vitamin D | rs10469075 | 64,979 | -0.032 | 0.007 | T | C | 21 |
|  | rs11255252 | 64,979 | 0.043 | 0.010 | A | G | 20 |
|  | rs117693112 | 64,979 | 0.068 | 0.014 | A | G | 22 |
|  | rs117732619 | 64,979 | -0.118 | 0.026 | C | T | 20 |
|  | rs12120559 | 64,979 | -0.025 | 0.005 | T | G | 20 |
|  | rs16959029 | 64,979 | 0.099 | 0.022 | G | A | 20 |
|  | rs17301981 | 64,979 | -0.044 | 0.010 | C | T | 21 |
|  | rs2399949 | 64,979 | -0.034 | 0.007 | C | T | 24 |
|  | rs35775421 | 64,979 | -0.056 | 0.012 | A | G | 21 |
|  | rs4395237 | 64,979 | -0.060 | 0.013 | G | T | 22 |
|  | rs57038272 | 64,979 | 0.033 | 0.007 | T | C | 21 |
|  | rs582962 | 64,979 | -0.028 | 0.006 | A | G | 22 |
|  | rs60858456 | 64,979 | 0.033 | 0.007 | C | T | 20 |
|  | rs61504980 | 64,979 | 0.029 | 0.006 | A | G | 20 |
|  | rs61942184 | 64,979 | 0.082 | 0.018 | C | G | 21 |
|  | rs679830 | 64,979 | -0.060 | 0.013 | C | T | 22 |
|  | rs72682991 | 64,979 | -0.037 | 0.008 | T | C | 20 |
|  | rs72899450 | 64,979 | 0.061 | 0.013 | A | G | 21 |
|  | rs73185990 | 64,979 | 0.103 | 0.023 | G | A | 20 |
|  | rs74593039 | 64,979 | 0.035 | 0.008 | C | G | 22 |
|  | rs75713989 | 64,979 | 0.039 | 0.008 | T | C | 23 |
|  | rs7705693 | 64,979 | -0.025 | 0.006 | T | C | 21 |
|  | rs80261862 | 64,979 | -0.045 | 0.009 | T | C | 23 |
|  | rs9561693 | 64,979 | -0.029 | 0.006 | T | C | 21 |
| Vitamin E | rs111306778 | 64,979 | -0.048 | 0.010 | A | G | 25 |
|  | rs112571059 | 64,979 | -0.065 | 0.015 | A | C | 20 |
|  | rs114524219 | 64,979 | 0.063 | 0.014 | A | G | 20 |
|  | rs115033309 | 64,979 | 0.091 | 0.020 | A | G | 20 |
|  | rs117519020 | 64,979 | -0.072 | 0.016 | G | A | 20 |
|  | rs11889555 | 64,979 | 0.028 | 0.006 | G | A | 20 |
|  | rs12128707 | 64,979 | 0.028 | 0.006 | G | A | 20 |
|  | rs12165526 | 64,979 | 0.048 | 0.009 | A | T | 27 |
|  | rs12421920 | 64,979 | -0.043 | 0.009 | G | A | 21 |
|  | rs12828066 | 64,979 | 0.027 | 0.006 | G | T | 20 |
|  | rs12899673 | 64,979 | 0.027 | 0.006 | A | G | 21 |
|  | rs148096533 | 64,979 | -0.099 | 0.022 | G | A | 20 |
|  | rs17242648 | 64,979 | -0.043 | 0.010 | T | C | 20 |
|  | rs188625854 | 64,979 | -0.102 | 0.023 | A | G | 20 |
|  | rs2036815 | 64,979 | 0.025 | 0.006 | T | C | 20 |
|  | rs2101142 | 64,979 | 0.066 | 0.015 | T | C | 20 |
|  | rs2723979 | 64,979 | -0.027 | 0.006 | G | T | 23 |
|  | rs35218694 | 64,979 | -0.074 | 0.015 | G | A | 23 |
|  | rs35992004 | 64,979 | -0.024 | 0.006 | A | G | 20 |
|  | rs480958 | 64,979 | 0.025 | 0.006 | A | G | 20 |
|  | rs4903544 | 64,979 | -0.030 | 0.006 | T | C | 24 |
|  | rs536912 | 64,979 | 0.030 | 0.006 | A | C | 24 |
|  | rs6033 | 64,979 | -0.052 | 0.011 | G | A | 24 |
|  | rs62347998 | 64,979 | 0.062 | 0.014 | T | C | 20 |
|  | rs71385328 | 64,979 | 0.130 | 0.026 | G | A | 25 |
|  | rs72757566 | 64,979 | -0.101 | 0.023 | A | T | 20 |
|  | rs7941633 | 64,979 | 0.033 | 0.007 | C | A | 20 |
|  | rs79966958 | 64,979 | -0.117 | 0.025 | T | C | 23 |
|  | rs80257223 | 64,979 | -0.036 | 0.008 | T | C | 20 |
|  | rs979218 | 64,979 | -0.043 | 0.009 | C | A | 22 |
|  | rs9801653 | 64,979 | 0.036 | 0.008 | A | C | 20 |
| Carotene | rs10278909 | 64,979 | 0.027 | 0.006 | G | A | 20 |
|  | rs10770208 | 64,979 | -0.033 | 0.007 | C | T | 20 |
|  | rs117731008 | 64,979 | 0.098 | 0.021 | A | G | 21 |
|  | rs12126792 | 64,979 | -0.135 | 0.028 | G | A | 23 |
|  | rs12463115 | 64,979 | -0.028 | 0.006 | T | C | 20 |
|  | rs13295574 | 64,979 | -0.028 | 0.006 | A | G | 21 |
|  | rs1689008 | 64,979 | -0.029 | 0.006 | A | C | 21 |
|  | rs16898247 | 64,979 | -0.107 | 0.020 | A | G | 29 |
|  | rs17800766 | 64,979 | -0.122 | 0.025 | C | T | 23 |
|  | rs1783730 | 64,979 | 0.025 | 0.006 | G | A | 20 |
|  | rs1936052 | 64,979 | -0.036 | 0.008 | T | C | 22 |
|  | rs2917604 | 64,979 | -0.029 | 0.006 | T | C | 20 |
|  | rs2998143 | 64,979 | -0.028 | 0.006 | G | A | 23 |
|  | rs366337 | 64,979 | 0.054 | 0.011 | G | A | 23 |
|  | rs3829931 | 64,979 | 0.083 | 0.018 | A | T | 22 |
|  | rs4771831 | 64,979 | -0.027 | 0.006 | A | G | 21 |
|  | rs5760695 | 64,979 | 0.047 | 0.010 | C | T | 22 |
|  | rs62075874 | 64,979 | 0.035 | 0.008 | C | A | 21 |
|  | rs62417408 | 64,979 | -0.069 | 0.015 | G | A | 22 |
|  | rs6596473 | 64,979 | 0.028 | 0.006 | C | G | 21 |
|  | rs6658791 | 64,979 | 0.053 | 0.012 | A | G | 20 |
|  | rs6660246 | 64,979 | -0.027 | 0.006 | C | A | 23 |
|  | rs73103257 | 64,979 | 0.037 | 0.008 | C | T | 21 |
|  | rs73213378 | 64,979 | 0.053 | 0.012 | T | C | 21 |
|  | rs76263256 | 64,979 | -0.110 | 0.024 | T | C | 20 |
|  | rs77547747 | 64,979 | -0.056 | 0.012 | C | T | 22 |
|  | rs77844870 | 64,979 | -0.057 | 0.013 | G | T | 20 |
|  | rs79407438 | 64,979 | 0.054 | 0.012 | T | C | 21 |
|  | rs806367 | 64,979 | -0.026 | 0.006 | C | T | 20 |
| Folate | rs113095798 | 64,979 | -0.077 | 0.017 | A | G | 20 |
|  | rs11673086 | 64,979 | 0.053 | 0.012 | A | G | 21 |
|  | rs12630658 | 64,979 | 0.025 | 0.006 | C | T | 20 |
|  | rs141864123 | 64,979 | 0.084 | 0.019 | A | G | 20 |
|  | rs148031795 | 64,979 | 0.104 | 0.022 | T | C | 22 |
|  | rs1502443 | 64,979 | 0.026 | 0.006 | G | C | 21 |
|  | rs16956822 | 64,979 | -0.079 | 0.017 | A | G | 21 |
|  | rs2198140 | 64,979 | 0.025 | 0.005 | C | T | 20 |
|  | rs2449166 | 64,979 | 0.025 | 0.005 | T | C | 21 |
|  | rs3772928 | 64,979 | -0.027 | 0.006 | C | T | 24 |
|  | rs45442894 | 64,979 | 0.110 | 0.024 | A | C | 21 |
|  | rs61868302 | 64,979 | -0.053 | 0.012 | T | C | 21 |
|  | rs633683 | 64,979 | 0.025 | 0.006 | C | T | 20 |
|  | rs7074988 | 64,979 | -0.051 | 0.011 | G | A | 21 |
|  | rs73173339 | 64,979 | -0.034 | 0.008 | G | A | 20 |
|  | rs76101819 | 64,979 | -0.094 | 0.021 | A | G | 20 |
|  | rs76630415 | 64,979 | -0.037 | 0.007 | G | T | 31 |
|  | rs76802001 | 64,979 | -0.068 | 0.015 | A | G | 21 |
|  | rs78074774 | 64,979 | 0.060 | 0.013 | T | C | 21 |
|  | rs79748722 | 64,979 | -0.076 | 0.016 | T | C | 21 |
|  | rs79975477 | 64,979 | 0.073 | 0.016 | T | C | 22 |
|  | rs8085166 | 64,979 | 0.028 | 0.006 | G | A | 23 |
| **Alzheimer's disease-Trace element** |  |  |  |  |  |  |  |
| Copper | rs10197620 | 184,190 | -1.046 | 0.229 | C | T | 21 |
|  | rs2528632 | 184,190 | 0.338 | 0.069 | G | A | 24 |
|  | rs970654 | 184,190 | -0.473 | 0.103 | A | G | 21 |
| Carotene | rs10197620 | 184,190 | -1.046 | 0.229 | C | T | 21 |
|  | rs112679245 | 184,190 | 0.729 | 0.153 | A | T | 23 |
|  | rs12145949 | 184,190 | 1.435 | 0.318 | A | G | 20 |
|  | rs141836872 | 184,190 | 1.713 | 0.372 | A | G | 21 |
|  | rs150963814 | 184,190 | 1.190 | 0.260 | A | C | 21 |
|  | rs2528632 | 184,190 | 0.338 | 0.069 | G | A | 24 |
|  | rs429358 | 184,190 | 1.076 | 0.074 | C | T | 210 |
|  | rs55846330 | 184,190 | 0.591 | 0.132 | C | T | 20 |
|  | rs5995691 | 184,190 | 0.348 | 0.070 | A | G | 25 |
|  | rs74549518 | 184,190 | 0.608 | 0.125 | A | G | 24 |
|  | rs80158324 | 184,190 | 1.127 | 0.235 | C | T | 23 |
|  | rs8113128 | 184,190 | 0.825 | 0.144 | C | A | 33 |
|  | rs970654 | 184,190 | -0.473 | 0.103 | A | G | 21 |
| **Trace element-Gut microbiota** |  |  |  |  |  |  |  |
| Copper-GCST90027490 | rs10014072 | 2,603 | -0.164 | 0.034 | G | A | 23 |
|  | rs1175550 | 2,603 | 0.198 | 0.032 | G | A | 38 |
|  | rs12521284 | 2,603 | -0.151 | 0.034 | T | A | 20 |
|  | rs13074172 | 2,603 | 0.121 | 0.027 | G | A | 20 |
|  | rs2769264 | 2,603 | 0.313 | 0.034 | G | T | 85 |
|  | rs3857536 | 2,603 | -0.129 | 0.028 | T | C | 21 |
|  | rs7206796 | 2,603 | -0.152 | 0.034 | T | C | 20 |
|  | rs764560 | 2,603 | -0.128 | 0.028 | T | C | 21 |
|  | rs9324493 | 2,603 | -0.177 | 0.039 | G | A | 21 |
| Carotene-GCST90027506 | rs10278909 | 64,979 | 0.027 | 0.006 | G | A | 20 |
|  | rs12463115 | 64,979 | -0.028 | 0.006 | T | C | 20 |
|  | rs1689008 | 64,979 | -0.029 | 0.006 | A | C | 21 |
|  | rs2917604 | 64,979 | -0.029 | 0.006 | T | C | 20 |
|  | rs4357613 | 64,979 | 0.026 | 0.006 | T | C | 22 |
|  | rs4771831 | 64,979 | -0.027 | 0.006 | A | G | 21 |
|  | rs5760694 | 64,979 | 0.047 | 0.010 | C | A | 22 |
|  | rs73103257 | 64,979 | 0.037 | 0.008 | C | T | 21 |
| Carotene-GCST90027699 | rs10278909 | 64,979 | 0.027 | 0.006 | G | A | 20 |
|  | rs12463115 | 64,979 | -0.028 | 0.006 | T | C | 20 |
|  | rs1689008 | 64,979 | -0.029 | 0.006 | A | C | 21 |
|  | rs2917604 | 64,979 | -0.029 | 0.006 | T | C | 20 |
|  | rs4357613 | 64,979 | 0.026 | 0.006 | T | C | 22 |
|  | rs4771831 | 64,979 | -0.027 | 0.006 | A | G | 21 |
|  | rs5760694 | 64,979 | 0.047 | 0.010 | C | A | 22 |
|  | rs73103257 | 64,979 | 0.037 | 0.008 | C | T | 21 |
| **Gut microbiota-Alzheimer's disease** |  |  |  |  |  |  |  |
| GCST90027490 | rs10918287 | 7,738 | -0.078 | 0.017 | A | G | 20 |
|  | rs11790347 | 7,738 | 0.172 | 0.038 | G | C | 21 |
|  | rs12677665 | 7,738 | 0.082 | 0.018 | G | A | 21 |
|  | rs1321819 | 7,738 | -0.084 | 0.018 | A | G | 21 |
|  | rs17118750 | 7,738 | 0.126 | 0.028 | C | T | 20 |
|  | rs2096694 | 7,738 | 0.087 | 0.018 | T | C | 23 |
|  | rs2244670 | 7,738 | -0.092 | 0.020 | A | T | 21 |
|  | rs62104460 | 7,738 | 0.126 | 0.028 | T | C | 20 |
|  | rs657516 | 7,738 | -0.157 | 0.035 | G | A | 20 |
|  | rs67156511 | 7,738 | -0.117 | 0.026 | G | T | 20 |
| GCST90027506 | rs12417998 | 7,738 | -0.130 | 0.029 | A | G | 20 |
|  | rs12599680 | 7,738 | -0.104 | 0.023 | T | C | 21 |
|  | rs12669414 | 7,738 | -0.079 | 0.017 | T | C | 23 |
|  | rs138494236 | 7,738 | -0.161 | 0.035 | C | T | 21 |
|  | rs140787604 | 7,738 | -0.172 | 0.039 | G | A | 20 |
|  | rs1486114 | 7,738 | -0.107 | 0.022 | G | T | 23 |
|  | rs17621104 | 7,738 | 0.114 | 0.025 | A | C | 21 |
|  | rs2038820 | 7,738 | -0.078 | 0.017 | C | T | 22 |
|  | rs4399424 | 7,738 | 0.081 | 0.018 | C | T | 20 |
|  | rs62074577 | 7,738 | 0.078 | 0.018 | G | A | 20 |
|  | rs7181967 | 7,738 | -0.096 | 0.020 | C | T | 22 |
|  | rs7228238 | 7,738 | -0.081 | 0.017 | T | C | 24 |
|  | rs7856581 | 7,738 | 0.171 | 0.035 | C | G | 24 |
| GCST90027699 | rs10760885 | 7,738 | 0.113 | 0.022 | A | T | 25 |
|  | rs115309509 | 7,738 | 0.219 | 0.049 | A | T | 20 |
|  | rs12895134 | 7,738 | 0.100 | 0.023 | A | G | 20 |
|  | rs246783 | 7,738 | 0.114 | 0.024 | G | A | 22 |
|  | rs2882286 | 7,738 | 0.142 | 0.030 | G | A | 22 |
|  | rs4767918 | 7,738 | 0.111 | 0.024 | C | T | 22 |
|  | rs61407789 | 7,738 | -0.202 | 0.046 | A | G | 20 |
|  | rs6465823 | 7,738 | 0.122 | 0.026 | T | G | 21 |
|  | rs704272 | 7,738 | -0.156 | 0.035 | C | T | 20 |
|  | rs72810711 | 7,738 | 0.114 | 0.026 | C | G | 20 |
|  | rs891501 | 7,738 | -0.157 | 0.033 | T | G | 23 |
| GCST90027490: NAGLIPASYN.PWY..lipid.IVA.biosynthesis; GCST90027506: PENTOSE.P.PWY..pentose.phosphate.pathway; GCST90027699: k_Bacteria.p_Bacteroidetes.c_Bacteroidia.o_Bacteroidales.f_Prevotellaceae.g_Paraprevotella | | | | | | | |

| **Table S3.** Mendelian randomization analysis and sensitivity analysis of genetic causality between trace element, gut microbiota and Alzheimer's disease | | | | | | | | |
| --- | --- | --- | --- | --- | --- | --- | --- | --- |
| **Exposure-Outcome** | **No. SNP** | **MR analysis** | | | **Heterogeneity** | | **Pleiotropy** | |
|  |  | **Methods** | **OR (95%CI)** | ***P*** | **Q** | ***P*** | **Intercept** | ***P*** |
| **Trace element-Alzheimer's disease** |  |  |  |  |  |  |  |  |
| Iron | 20 | MR Egger | 2.902(0.126-66.620) | 5.137E-01 | 18 | 0.724 |  |  |
|  |  | WM | 0.599(0.120-2.999) | 5.333E-01 |  |  |  |  |
|  |  | IVW | 1.159(0.362-3.716) | 8.037E-01 | 19 | 0.756 | -0.037 | 0.544 |
|  |  | Simple mode | 0.411(0.030-5.651) | 5.138E-01 |  |  |  |  |
|  |  | Weighted mode | 0.360(0.029-4.486) | 4.372E-01 |  |  |  |  |
| Sodium | 54 | MR Egger | 0.494(0.013-18.732) | 7.050E-01 | 52 | 0.212 |  |  |
|  |  | WM | 1.164(0.323-4.190) | 8.160E-01 |  |  |  |  |
|  |  | IVW | 1.198(0.462-3.104) | 7.105E-01 | 53 | 0.234 | 0.024 | 0.622 |
|  |  | Simple mode | 1.614(0.123-21.235) | 7.174E-01 |  |  |  |  |
|  |  | Weighted mode | 1.271(0.152-10.609) | 8.255E-01 |  |  |  |  |
| Potassium | 31 | MR Egger | 0.308(0.014-6.933) | 4.646E-01 | 29 | 0.318 |  |  |
|  |  | WM | 2.407(0.561-10.328) | 2.372E-01 |  |  |  |  |
|  |  | IVW | 1.731(0.613-4.883) | 3.000E-01 | 30 | 0.301 | 0.065 | 0.259 |
|  |  | Simple mode | 6.158(0.295-128.703) | 2.504E-01 |  |  |  |  |
|  |  | Weighted mode | 6.158(0.333-114.010) | 2.317E-01 |  |  |  |  |
| Zinc | 13 | MR Egger | 0.945(0.342-2.609) | 9.153E-01 | 11 | 0.073 |  |  |
|  |  | WM | 0.899(1.034-1.974) | 1.536E-01 |  |  |  |  |
|  |  | IVW | 1.269(0.903-1.784) | 1.692E-01 | 12 | 0.088 | 0.064 | 0.557 |
|  |  | Simple mode | 0.836(0.388-1.800) | 6.553E-01 |  |  |  |  |
|  |  | Weighted mode | 1.350(0.731-2.494) | 3.566E-01 |  |  |  |  |
| Copper | 11 | MR Egger | 1.318(1.016-1.710) | 6.701E-02 | 9 | 0.735 |  |  |
|  |  | WM | 1.301(1.008-1.678) | **4.336E-02** |  |  |  |  |
|  |  | IVW | 1.291(1.075-1.551) | **6.352E-03** | 10 | 0.807 | -0.008 | 0.830 |
|  |  | Simple mode | 1.517(0.978-2.353) | 9.256E-02 |  |  |  |  |
|  |  | Weighted mode | 1.334(1.055-1.686) | **3.678E-02** |  |  |  |  |
| Calcium | 27 | MR Egger | 0.257(0.009-7.422) | 4.360E-01 | 25 | 0.295 |  |  |
|  |  | WM | 0.456(0.098-2.114) | 3.154E-01 |  |  |  |  |
|  |  | IVW | 0.629(0.206-1.926) | 4.173E-01 | 26 | 0.328 | 0.034 | 0.584 |
|  |  | Simple mode | 0.179(0.010-3.275) | 2.567E-01 |  |  |  |  |
|  |  | Weighted mode | 0.312(0.878-5.04) | 4.198E-01 |  |  |  |  |
| Phosphorus | 16 | MR Egger | 5.260(0.623-44.383) | 1.493E-01 | 14 | 0.219 |  |  |
|  |  | WM | 1.106(0.413-2.956) | 8.415E-01 |  |  |  |  |
|  |  | IVW | 0.711(0.284-1.778) | 4.659E-01 | 15 | 0.089 | -0.126 | 0.065 |
|  |  | Simple mode | 0.745(0.176-3.145) | 6.940E-01 |  |  |  |  |
|  |  | Weighted mode | 1.167(0.450-3.024) | 7.551E-01 |  |  |  |  |
| Selenium | 14 | MR Egger | 0.782(0.503-1.216) | 2.971E-01 | 12 | 0.262 |  |  |
|  |  | WM | 0.936(0.682-1.285) | 6.844E-01 |  |  |  |  |
|  |  | IVW | 1.086(0.836-1.409) | 5.374E-01 | 13 | 0.145 | 0.082 | 0.107 |
|  |  | Simple mode | 1.440(0.840-2.471) | 2.081E-01 |  |  |  |  |
|  |  | Weighted mode | 0.851(0.598-1.212) | 3.879E-01 |  |  |  |  |
| Magnesium | 32 | MR Egger | 0.612(0.075-4.970) | 6.494E-01 | 30 | 0.214 |  |  |
|  |  | WM | 0.723(0.185-2.824) | 6.407E-01 |  |  |  |  |
|  |  | IVW | 0.813(0.298-2.216) | 6.861E-01 | 31 | 0.249 | 0.014 | 0.763 |
|  |  | Simple mode | 0.341(0.025-4.719) | 4.282E-01 |  |  |  |  |
|  |  | Weighted mode | 0.876(0.091-8.461) | 9.094E-01 |  |  |  |  |
| Vitamin A | 17 | MR Egger | 5.028e+20(3.697e-34-6.838e+74) | 4.651E-01 | 15 | 0.074 |  |  |
|  |  | WM | 1.027e+16(0.215-4.912e+32) | 5.991E-02 |  |  |  |  |
|  |  | IVW | 1.739e+7(6.724e-7-4.496e+20) | 2.900E-01 | 16 | 0.092 | -0.061 | 0.622 |
|  |  | Simple mode | 1.019e+27(0.014-7.265e+55) | 8.521E-02 |  |  |  |  |
|  |  | Weighted mode | 1.019e+27(0.010-1.082e+56) | 8.692E-02 |  |  |  |  |
| Vitamin B6 | 30 | MR Egger | 1.269(0.132-12.221) | 8.381E-01 | 28 | 0.375 |  |  |
|  |  | WM | 0.224(0.058-0.868) | **3.039E-02** |  |  |  |  |
|  |  | IVW | 0.491(0.180-1.336) | 1.635E-01 | 29 | 0.382 | -0.042 | 0.367 |
|  |  | Simple mode | 0.125(0.009-1.725) | 1.312E-01 |  |  |  |  |
|  |  | Weighted mode | 0.175(0.016-1.883) | 1.612E-01 |  |  |  |  |
| Vitamin B12 | 25 | MR Egger | 0.528(0.049-5.640) | 6.021E-01 | 23 | 0.667 |  |  |
|  |  | WM | 0.621(0.151-2.556) | 5.091E-01 |  |  |  |  |
|  |  | IVW | 1.212(0.443-3.316) | 7.077E-01 | 24 | 0.688 | 0.035 | 0.455 |
|  |  | Simple mode | 0.354(0.020-6.416) | 4.891E-01 |  |  |  |  |
|  |  | Weighted mode | 0.335(0.018-6.191) | 4.694E-01 |  |  |  |  |
| Vitamin C | 22 | MR Egger | 3.744(0.125-112.142) | 4.555E-01 | 20 | 0.043 |  |  |
|  |  | WM | 7.789(1.388-43.696) | **1.966E-02** |  |  |  |  |
|  |  | IVW | 3.967(0.984-15.984) | 5.264E-02 | 21 | 0.058 | 0.003 | 0.971 |
|  |  | Simple mode | 15.652(0.630-388.615) | 1.081E-01 |  |  |  |  |
|  |  | Weighted mode | 9.155(0.343-244.378) | 2.006E-01 |  |  |  |  |
| Vitamin D | 24 | MR Egger | 0.286(0.017-4.881) | 3.967E-01 | 22 | 0.605 |  |  |
|  |  | WM | 1.468(0.312-6.905) | 6.267E-01 |  |  |  |  |
|  |  | IVW | 1.433(0.494-4.159) | 5.085E-01 | 23 | 0.575 | 0.069 | 0.243 |
|  |  | Simple mode | 0.872(0.060-12.606) | 9.206E-01 |  |  |  |  |
|  |  | Weighted mode | 1.275(0.102-15.941) | 8.520E-01 |  |  |  |  |
| Vitamin E | 31 | MR Egger | 1.750(0.199-15.385) | 6.177E-01 | 29 | 0.283 |  |  |
|  |  | WM | 0.862(0.218-3.412) | 8.322E-01 |  |  |  |  |
|  |  | IVW | 0.948(0.353-2.545) | 9.161E-01 | 30 | 0.309 | -0.028 | 0.539 |
|  |  | Simple mode | 0.624(0.054-7.185) | 7.080E-01 |  |  |  |  |
|  |  | Weighted mode | 1.287(0.218-7.591) | 7.826E-01 |  |  |  |  |
| Carotene | 29 | MR Egger | 1.646(0.142-19.087) | 6.934E-01 | 27 | 0.914 |  |  |
|  |  | WM | 3.556(0.965-13.105) | 5.663E-02 |  |  |  |  |
|  |  | IVW | 2.805(1.054-7.466) | **3.893E-02** | 28 | 0.930 | 0.022 | 0.646 |
|  |  | Simple mode | 3.370(0.327-34.748) | 3.163E-01 |  |  |  |  |
|  |  | Weighted mode | 3.901(0.404-37.628) | 2.490E-01 |  |  |  |  |
| Folate | 22 | MR Egger | 4.712(0.394-56.357) | 2.351E-01 | 20 | 0.597 |  |  |
|  |  | WM | 2.833(0.514-15.611) | 2.316E-01 |  |  |  |  |
|  |  | IVW | 1.821(0.573-5.789) | 3.095E-01 | 21 | 0.613 | -0.042 | 0.406 |
|  |  | Simple mode | 6.210(0.295-130.550) | 2.531E-01 |  |  |  |  |
|  |  | Weighted mode | 5.980(0.371-96.345) | 2.211E-01 |  |  |  |  |
| **Alzheimer's disease-Trace element** |  |  |  |  |  |  |  |  |
| Copper | 3 | MR Egger | 0.929(0.766-1.127) | 5.918E-01 |  |  |  |  |
|  |  | WM | 1.004(0.917-1.100) | 9.274E-01 |  |  |  |  |
|  |  | IVW | 0.995(0.918-1.079) | 9.049E-01 |  |  |  |  |
|  |  | Simple mode | 1.028(0.919-1.150) | 6.785E-01 |  |  |  |  |
|  |  | Weighted mode | 0.958(0.861-1.067) | 5.204E-01 |  |  |  |  |
| Carotene | 12 | MR Egger | 1.014(0.997-1.030) | 1.419E-01 |  |  |  |  |
|  |  | WM | 0.997(0.986-1.008) | 5.922E-01 |  |  |  |  |
|  |  | IVW | 0.998(0.989-1.006) | 5.921E-01 |  |  |  |  |
|  |  | Simple mode | 0.989(0.970-1.008) | 2.849E-01 |  |  |  |  |
|  |  | Weighted mode | 0.995(0.982-1.008) | 4.841E-01 |  |  |  |  |
| **Trace element-Gut microbiota** |  |  |  |  |  |  |  |  |
| Copper-GCST90027490 | 9 | MR Egger | 1.204(0.956-1.517) | 1.591E-01 | 7 | 0.916 |  |  |
|  |  | WM | 1.150(1.046-1.264) | **3.710E-03** |  |  |  |  |
|  |  | IVW | 1.125(1.046-1.210) | **1.530E-03** | 8 | 0.934 | -0.012 | 0.562 |
|  |  | Simple mode | 1.093(0.954-1.252) | 2.355E-01 |  |  |  |  |
|  |  | Weighted mode | 1.148(1.032-1.277) | **3.513E-02** |  |  |  |  |
| Carotene-GCST90027506 | 8 | MR Egger | 1.038(0.070-15.370) | 9.793E-01 | 6 | 0.629 |  |  |
|  |  | WM | 0.656(0.368-1.171) | 1.538E-01 |  |  |  |  |
|  |  | IVW | 0.593(0.374-0.939) | **2.571E-02** | 7 | 0.718 | -0.017 | 0.694 |
|  |  | Simple mode | 0.657(0.257-1.677) | 4.084E-01 |  |  |  |  |
|  |  | Weighted mode | 0.674(0.275-1.649) | 4.158E-01 |  |  |  |  |
| Carotene-GCST90027699 | 8 | MR Egger | 0.261(0.007-10.462) | 5.027E-01 | 6 | 0.630 |  |  |
|  |  | WM | 0.543(0.244-1.209) | 1.348E-01 |  |  |  |  |
|  |  | IVW | 0.530(0.281-0.999) | **4.976E-02** | 7 | 0.722 | 0.021 | 0.716 |
|  |  | Simple mode | 0.478(0.129-1.775) | 3.066E-01 |  |  |  |  |
|  |  | Weighted mode | 0.478(0.137-1.664) | 2.841E-01 |  |  |  |  |
| **Gut microbiota-Alzheimer's disease** |  |  |  |  |  |  |  |  |
| GCST90027490 | 10 | MR Egger | 1.761(0.185-16.749) | 6.355E-01 | 8 | 0.748 |  |  |
|  |  | WM | 2.263(1.096-4.673) | **2.731E-02** |  |  |  |  |
|  |  | IVW | 1.822(1.036-3.206) | **3.734E-02** | 9 | 0.827 | 0.004 | 0.976 |
|  |  | Simple mode | 2.512(0.879-7.181) | 1.198E-01 |  |  |  |  |
|  |  | Weighted mode | 2.512(0.864-7.301) | 1.249E-01 |  |  |  |  |
| GCST90027506 | 13 | MR Egger | 0.468(0.080-2.730) | 4.171E-01 | 11 | 0.822 |  |  |
|  |  | WM | 0.476(0.247-0.918) | **2.668E-02** |  |  |  |  |
|  |  | IVW | 0.604(0.375-0.974) | **3.868E-02** | 12 | 0.870 | 0.027 | 0.774 |
|  |  | Simple mode | 0.410(0.146-1.149) | 1.158E-01 |  |  |  |  |
|  |  | Weighted mode | 0.401(0.140-1.148) | 1.143E-01 |  |  |  |  |
| GCST90027699 | 10 | MR Egger | 0.731(0.125-4.285) | 7.369E-01 | 8 | 0.971 |  |  |
|  |  | WM | 0.576(0.338-0.983) | **4.291E-02** |  |  |  |  |
|  |  | IVW | 0.593(0.389-0.906) | **1.561E-02** | 9 | 0.985 | -0.029 | 0.818 |
|  |  | Simple mode | 0.618(0.264-1.450) | 2.974E-01 |  |  |  |  |
|  |  | Weighted mode | 0.598(0.257-1.392) | 2.633E-01 |  |  |  |  |
| MR: Mendelian randomization; WM: Weighted median; IVW: Inverse variance weighted; GCST90027490: NAGLIPASYN.PWY..lipid.IVA.biosynthesis; GCST90027506: PENTOSE.P.PWY..pentose.phosphate.pathway; GCST90027699: k_Bacteria.p_Bacteroidetes.c_Bacteroidia.o_Bacteroidales.f_Prevotellaceae.g_Paraprevotella | | | | | | | | |
